# Calculating variant penetrance from family history of disease and average family size in population-scale data

**DOI:** 10.1186/s13073-022-01142-7

**Published:** 2022-12-15

**Authors:** Thomas P. Spargo, Sarah Opie-Martin, Harry Bowles, Cathryn M. Lewis, Alfredo Iacoangeli, Ammar Al-Chalabi

**Affiliations:** 1 Department of Basic and Clinical Neuroscience, Maurice Wohl Clinical Neuroscience Institute, King’s College London, London, SE5 9RX UK; 2 Social, Genetic and Developmental Psychiatry Centre, Institute of Psychiatry, Psychology & Neuroscience, King’s College London, de Crespigny Park, London, SE5 8AF UK; 3 Department of Medical and Molecular Genetics, Faculty of Life Sciences and Medicine, King’s College London, London, UK; 4 Department of Biostatistics and Health Informatics, King’s College London, London, UK; 5 NIHR Maudsley Biomedical Research Centre (BRC) at South London and Maudsley NHS Foundation Trust and King’s College London, London, UK; 6 King’s College Hospital, Bessemer Road, London, SE5 9RS UK

**Keywords:** Penetrance, Variant, Autosomal dominant, Disease, Familial, Sporadic

## Abstract

**Background:**

Genetic penetrance is the probability of a phenotype when harbouring a particular pathogenic variant. Accurate penetrance estimates are important across biomedical fields including genetic counselling, disease research, and gene therapy. However, existing approaches for penetrance estimation require, for instance, large family pedigrees or availability of large databases of people affected and not affected by a disease.

**Methods:**

We present a method for penetrance estimation in autosomal dominant phenotypes. It examines the distribution of a variant among people affected (cases) and unaffected (controls) by a phenotype within population-scale data and can be operated using cases only by considering family disease history. It is validated through simulation studies and candidate variant-disease case studies.

**Results:**

Our method yields penetrance estimates which align with those obtained via existing approaches in the Parkinson’s disease *LRRK2* gene and pulmonary arterial hypertension *BMPR2* gene case studies. In the amyotrophic lateral sclerosis case studies, examining penetrance for variants in the *SOD1* and *C9orf72* genes, we make novel penetrance estimates which correspond closely to understanding of the disease.

**Conclusions:**

The present approach broadens the spectrum of traits for which reliable penetrance estimates can be obtained. It has substantial utility for facilitating the characterisation of disease risks associated with rare variants with an autosomal dominant inheritance pattern. The yielded estimates avoid any kinship-specific effects and can circumvent ascertainment biases common when sampling rare variants among control populations.

**Supplementary Information:**

The online version contains supplementary material available at 10.1186/s13073-022-01142-7.

## Background

Penetrance is the probability of developing a specific trait given a genetic variant or set of variants. Some pathogenic variants are fully penetrant, and people harbouring them always develop the associated phenotype. For instance, a trinucleotide CAG repeat expansion within the *HTT* gene [MIM: 613004] is fully penetrant for Huntington’s Disease [MIM: 143100] by 80 years of age among people harbouring an expansion variant larger than 41 repeats [1]. For many variants, however, penetrance is incomplete, and those with risk variants can remain unaffected throughout their life. For example, the p.Gly2019Ser (c.6055G > A) variant of the *LRRK2* gene [MIM: 609007] exhibits incomplete penetrance for Parkinson’s disease (PD [MIM: 168600]), meaning that it elevates risk for PD but does not necessarily result in its manifestation [2].

In medical genetics, estimating the penetrance of pathogenic variants is vital for the correct interpretation of genetic test results. This importance will increase as genome sequencing becomes routine, both within and outside clinical practice, alongside advancements in precision medicine and gene therapy [3–6].

Several methods exist for penetrance estimation. The first and most widely used is based on statistical examination of how the variant segregates with the phenotype within pedigrees [7]. However, the generalisability of estimates derived from specific families may be limited. Other approaches examine the incidence of disease in a sample of unrelated people who harbour a variant [8, 9]. Without systematic sampling, these estimates can be affected by ascertainment bias. Where large pedigrees are not available, or if the disease is rare or late onset, these techniques may not be possible [10].

Estimating penetrance for a variant of unknown significance identified, for example, during genome sequencing-based screening can be particularly challenging. The problem is exemplified by the large number of reported *SOD1* gene [MIM: 147450] variants implicated in amyotrophic lateral sclerosis (ALS [MIM: 105400]). ALS is a fatal neurodegenerative disease characterised predominantly by progressive degeneration of motor neurons [11, 12]. *SOD1* variants are an important cause of ALS and over 180 ALS-associated variants in this gene have been reported to date [11, 13–15], however, family pedigrees suitable for establishing penetrance are available for only a minority of these.

We have developed a new method to estimate penetrance for variants with an autosomal dominant inheritance pattern using population-level data from unrelated people who are and are not affected by the associated phenotype. It can be operated using variant information drawn only from affected populations, stratified according to the family history between ‘familial’ and ‘sporadic’ disease presentations. This approach is based on our previously published model of disease which explains how variant penetrance and sibship size determine the presence or absence of a disease for families in which the variant occurs [16].

The method is complementary to and fills an important gap left by existing techniques. Using population-scale data, it takes full advantage of the rapidly growing quantity of genetic data that are being generated for a wide range of human disease and therefore it is ideally placed to be a valuable tool in the precision medicine era. Moreover, the capacity to assess penetrance based on the distribution of a variant between samples of unrelated people drawn only from the affected population allows estimates unbiased by kinship-specific effects or ascertainment of unaffected population members.

We have tested the approach in four variant-disease case studies, drawing upon the most common and widely studied autosomal dominant variants implicated in each disease: the *LRRK2* p.Gly2019Ser variant for PD [2]; variants in the *BMPR2* gene [MIM: 600799] for heritable pulmonary arterial hypertension (PAH [MIM: 178600]) [17]; and variants in the *SOD1* and *C9orf72* [MIM: 614260] genes for ALS [11, 12].

## Methods

### Model

Here, we describe an approach to estimate genetic penetrance for autosomal dominant traits using population-scale data.

Our method builds upon and extends an existing disease model [16] which makes the following assumptions: in a nuclear family, a rare dominant pathogenic variant is necessary but not sufficient for disease to occur, therefore penetrance, denoted $f$, is not complete and family members who do not harbour the variant are not affected; all variants are inherited from exactly one parent, thus there are no people homozygous for the variant or de novo variants. Our extended model relaxes the assumption that the variant is necessary for disease to occur: it assumes that people not harbouring the variant have a residual risk for developing disease after accounting for the proportion of disease occurrences attributed to the variant, denoted $g$.

Accordingly, if the probability of an individual being affected by a disease, $P(A)$, is $f$ when harbouring variant $M$ or $g$ if $M$ is absent, denoted ${M}^{^{\prime}}$, $P(A)$ can be determined by considering the probability of harbouring $M$, $P(M)$:

1 $$P\left(A\right)=f\times P\left(M\right)+g\times P\left({M}^{^{\prime}}\right),$$

letting $$P\left(M\mathrm{^{\prime}}\right)=1-P\left(M\right)$$.

In a family where a single parent harbours, and each child has a 0.5 chance of inheriting, $M$, the following probabilities of being affected can be determined per family member:

2 $$P{\left(A\right)}^{M}=f$$

for the variant harbouring parent, where $P(M)=1$;

3 $$P{\left(A\right)}^{{M}^{0.5}}=\frac{f}{2}+\frac{g}{2}$$

for each offspring, each of whom has $P(M)=0.5$, and thus risk influenced by both $f$ and $g$; and

4 $$P{\left(A\right)}^{{M}^{^{\prime}}}=g$$

for the parent without $M$, where $P(M)=0$, and therefore for whom disease risk is only determined by that which is associated with ${M}^{^{\prime}}$.

Considering these individual disease probabilities, three probabilities can be determined for a nuclear family where one parent harbours a given variant: that no family members are affected, $P(unaffected)$; that exactly one member is affected, $P(sporadic)$; and that more than one member is affected, $P(familial)$. These probabilities are determined by penetrance, $f$, residual disease risk $g$ if not harbouring the variant, and sibship size, $N$. In a family with $N$ siblings:

5 $$P\left(unaffected\right)=\left(1-f\right){\left(1-\frac{f}{2}-\frac{g}{2}\right)}^{N}\left(1-g\right) ,$$

where no family member, with or without the variant, develops the disease, and where each of the sibs has ${}^{1}\!\left/ \!{}_{2}\right.$ probability of being transmitted the variant.

6 $$P\left(sporadic\right)={f\left(1-\frac{f}{2}-\frac{g}{2}\right)}^{N}\left(1-g\right)+\left(1-f\right)N\left(\frac{f}{2}+\frac{g}{2}\right){\left(1-\frac{f}{2}-\frac{g}{2}\right)}^{N-1}\left(1-g\right)+(1-{f)\left(1-\frac{f}{2}-\frac{g}{2}\right)}^{N}g ,$$

if one family member develops the disease. This may be either may be the variant-harbouring parent, exactly one of the sibs, or the parent not harbouring the variant (on account of residual risk $g$). Then,

7 $$P\left(familial\right)=1-P\left(unaffected\right)-P\left(sporadic\right)=1-\left(\begin{array}{c}\left(1-f\right){\left(1-\frac{f}{2}-\frac{g}{2}\right)}^{N}\left(1-g\right)+\\ {f\left(1-\frac{f}{2}-\frac{g}{2}\right)}^{N}\left(1-g\right)+\\ \left(1-f\right)N\left(\frac{f}{2}+\frac{g}{2}\right){\left(1-\frac{f}{2}-\frac{g}{2}\right)}^{N-1}\left(1-g\right)+\\ (1-{f)\left(1-\frac{f}{2}-\frac{g}{2}\right)}^{N}g\end{array}\right) ,$$

where two or more family members develop the disease, which can be determined from $P(unaffected)$ and $P(sporadic)$ since the total probability of a family being unaffected, sporadic, or familial must sum to 1.

If $g=0$, the original [16] and extended models are equivalent.

### Application to penetrance calculation

Conversely, penetrance can be estimated given the observed rates of the unaffected, sporadic, and familial disease states in families where the pathogenic variant occurs, the average sibship size for these families, and an estimate of residual disease risk $g$. We can also estimate penetrance based on the observed rates of families presenting as unaffected versus *affected*, a fourth disease state whereby

8 $$P\left(affected\right)=P\left(familial\right)+P\left(sporadic\right) .$$

The observed rate of the arbitrarily-labelled disease state ‘X’, $R{\left(X\right)}^{obs}$, is used to indicate the frequency of one of the sampled disease states across all states sampled. $R{\left(X\right)}^{obs}$ can be specified for any valid combination of the four disease states, drawing from any two or three of the familial, sporadic, and unaffected disease states, or from the affected and unaffected states. Data from the affected state cannot be specified alongside that of the familial or sporadic disease states since the former is determined through their combination. $R{\left(X\right)}^{obs}$ may be specified directly if the distribution of disease states across people with the variant is known for the state-combination used or derived as a weighted proportion of estimates of heterozygous variant frequency across people with and without the variant (see Table 1).

**Table 1:** Valid disease state combinations and corresponding weighting factors for estimating disease state rates

Variant frequencies provided	Required weighting factors
Familial (${M}_{F}$), Sporadic (${M}_{S}$)	${W}_{F}=P\left(F|A\right)$, ${W}_{S}=P(S|A)$
Familial (${M}_{F}$), Unaffected (${M}_{U}$)	${W}_{F}=P(F|A)\times P{\left(A\right)}^{pop}$, ${W}_{U}=1-P{\left(A\right)}^{pop}$
Sporadic (${M}_{S}$), Unaffected (${M}_{U}$)	${W}_{S}=P\left(S|A\right)\times P{\left(A\right)}^{pop}$, ${W}_{U} =1-P{\left(A\right)}^{pop}$
Familial (${M}_{F}$), Sporadic (${M}_{S}$), Unaffected (${M}_{U}$)	${W}_{F}=P(F|A)\times P{\left(A\right)}^{pop}$, ${W}_{S}=P(S|A)\times P{\left(A\right)}^{pop}$, ${W}_{U}=1-P{\left(A\right)}^{pop}$
Affected (${M}_{A}$), Unaffected (${M}_{U}$)	${W}_{A}=P{\left(A\right)}^{pop}$, ${W}_{U}=1-P{\left(A\right)}^{pop}$

**Fig. 1:**
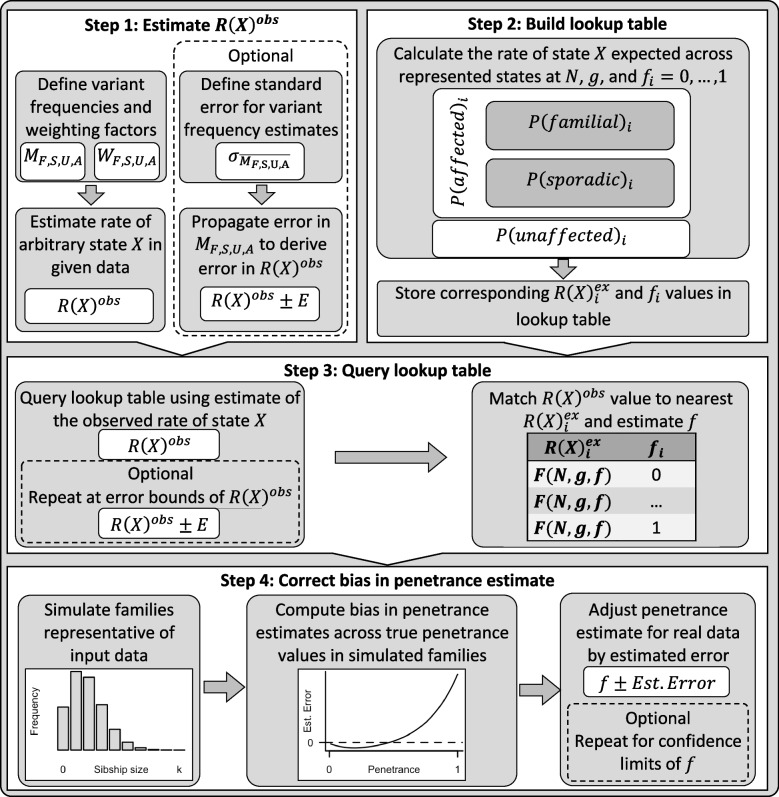
Summary of the key steps within this penetrance estimation approach. Legend: Step 1: Variant frequencies (M) and weighting factors (W) are defined for a valid subset of the familial (F), sporadic (S), unaffected (U), and affected (A) states (see Table 1) to calculate rate of one of these states, arbitrarily labelled state X, among families harbouring the pathogenic variant across those states with data provided, $R{\left(X\right)}^{obs}$. Step 2: Eqs. (5–8) are applied to calculate $P(familial)$, $P(sporadic)$, $P(unaffected)$, and $P(affected)$, for a series of penetrance values, $f_{i}=0,\dots ,1$, at a defined sibship size, $N$, and with disease risk $g$ for people not harbouring the variant. The rate of state X expected at each $f_{i}$ among variant harbouring families from those states represented in Step 1, $R{\left(X\right)}_{i}^{ex}$, is calculated and stored alongside the corresponding $f_{i}$ in a lookup table. Step 3: The lookup table is queried using $R{\left(X\right)}^{obs}$ to identify the closest $R{\left(X\right)}_{i}^{ex}$ value and corresponding $f_{i}$. Step 4: Bias in the obtained $f_{i}$ estimate is corrected by simulating a population of families representative of the sample data, estimating the difference between true and estimated penetrance values in this population between $f=0,\dots ,1$ and adjusting the estimated $f_{i}$ by error predicted within a polynomial regression model fitted upon the simulated estimate errors. Optional step: Confidence intervals for $R{\left(X\right)}^{obs}$ can be calculated from error in the estimates of $M$ provided [48]; Penetrance is estimated as in Steps 3 and 4 for the interval bounds. All steps within this approach are comprehensively detailed in Additional File: Sect. 1.1

The defined weighting factors are used in Step 1 of the penetrance estimation approach, as described in Fig. 1 and Additional File 1: Sect. 1.1$M_{F,S,U,A}$= variant frequencies in the familial, sporadic, unaffected, and affected states;$W_{F,S,U,A}$= weighting factors for the familial, sporadic, unaffected, and affected states;$P{\left(A\right)}^{pop}$= the probability of a member of the sampled population being affected;$P(F|A)$= disease familiality rate;$P(S|A)$= disease sporadic rate

Sibship size can be estimated for the sample either directly, based on the average sibship size among sampled families, or indirectly, by designating an estimate representative of the sample (e.g. from global databases).

Under Bayes theorem [18], $g$ can be determined from $P(A)$ and $P(M)$ within the general population, respectively $P{\left(A\right)}^{pop}$ and $P{\left(M\right)}^{pop}$, and the frequency of variant $M$ among people affected by disease, ${M}_{A}$:

9 $$g=\frac{P{\left(A\right)}^{pop}\times \left(1-{M}_{A}\right)}{\left(1-P{\left(M\right)}^{pop}\right)}.$$

${M}_{A}$ and $P{\left(M\right)}^{pop}$ may each be determined by weighted sums:

10 $${M}_{A}={M}_{F}\times P\left(F|A\right)+{M}_{S}\times P\left(S|A\right) ,$$

and

11 $$P{\left(M\right)}^{pop}={M}_{A}\times P{\left(A\right)}^{pop}+{M}_{U}\times \left(1-P{\left(A\right)}^{pop}\right) ,$$

where ${M}_{F,S,U}$ denote the variant frequencies in the familial, sporadic, and unaffected states, $P(F|A)$ is the rate at which people in the affected population, $A$, are familial, and $P(S|A)$ is the disease sporadic rate ($P(S|A)=1-P(F|A)$). If the disease is rare in the population, $g\approx 0$ and has a negligible influence upon penetrance estimates (see the simulation studies in Additional File 1: Sect. 1.2.3).

Our penetrance calculation method involves four steps and includes the option to derive error in the estimate. These processes are summarised in Fig. 1 and comprehensively outlined in Additional File 1: Sect. 1.1.

The method assumes that: one person is sampled per family and disease states are assigned based on the status of the person sampled and first-degree family members only; all variants are inherited from exactly one parent and there are no de novo variants; the value specified for sibship size is representative of sibship size across disease state groups. We recommend providing an estimate of $g$, however, $g=0$ by default, which makes the additional assumption that the trait only occurs in members of sampled families owing to the presence of the variant.

A further assumption is made in each of the two scenarios for determining $R{\left(X\right)}^{obs}$. When sampling across only families where the variant occurs, it is assumed that disease state classifications for sampled families will not change at a future time. When estimating variant frequencies within disease states across cohorts of people with and without the variant, it is assumed that family disease states change comparably over time for people with and without the variant. The latter assumption can be partially tested by examining whether age of disease onset is equally variable for people with and without the tested variant; the assumption is further discussed in Additional File 1: Sect. 1.2.2.

Additional File: Sect. 1.2 outlines the steps taken for approach validation, including details of several simulation studies and comparison between using a lookup table or maximum-likelihood approach for Step 3. The included simulation studies test accuracy in penetrance estimation when input parameters are correctly or incorrectly specified, when $g$ is accurately measured or assumed to be 0, and according to age of sampling across several scenarios.

**Fig. 2:**
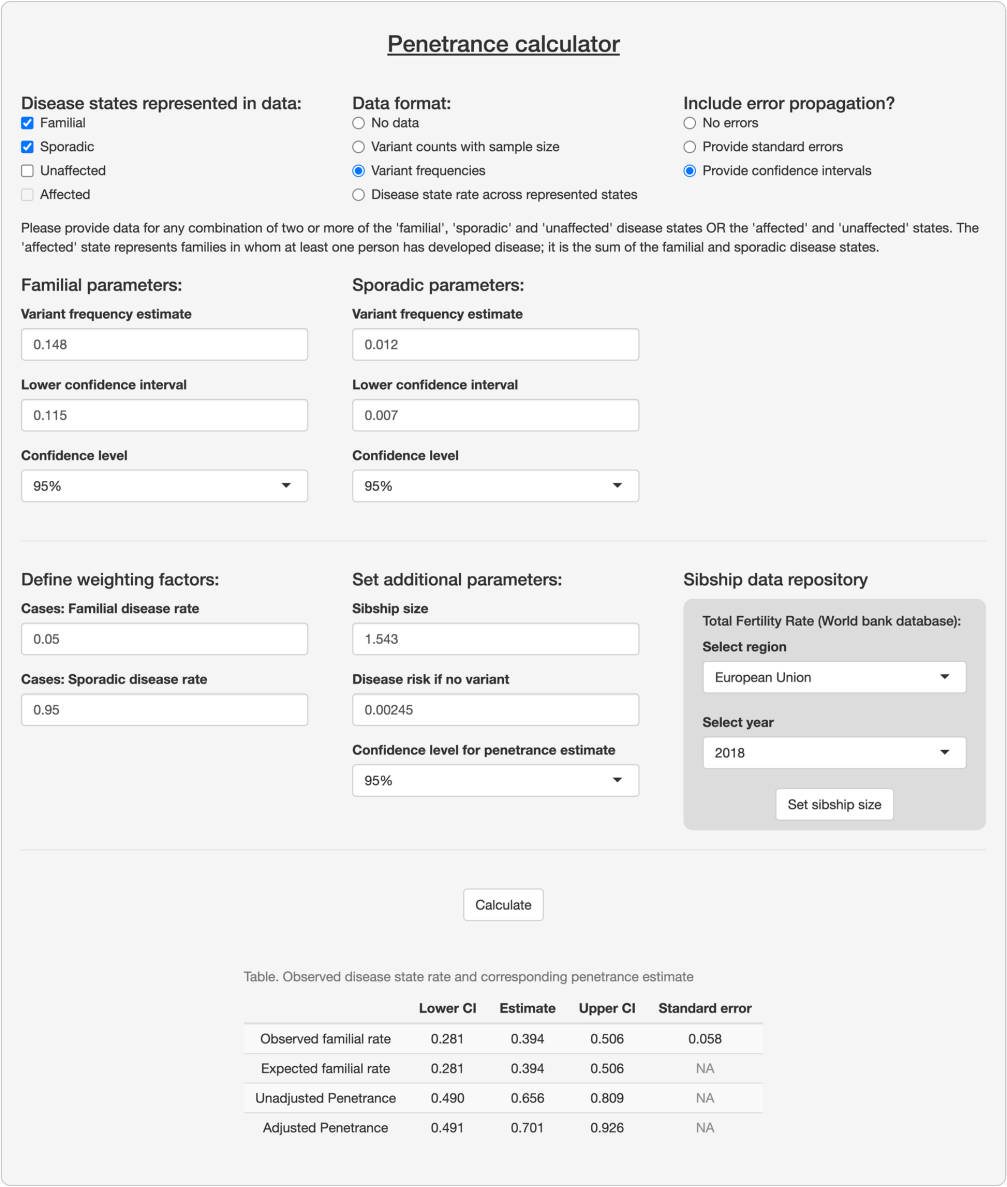
Example interface and output of the ADPenetrance web tool [20]. Legend: Here we show the example of penetrance of SOD1 variants for amyotrophic lateral sclerosis in a European population, applying variant frequency estimates for familial and sporadic ALS patients of European ancestry, an estimate of ALS disease risk among people not harbouring SOD1 variants, and the average Total Fertility Rate for the European Union in 2018 [22, 35]

We have made this approach available as the R function *adpenetrance* hosted on GitHub [19]. In the GitHub repository, we additionally provide functions to calculate $g$; test for equal onset variability across two groups; and simulate how a certain degree of unequal onset variability, as indicated by the previous function, may affect penetrance estimates. To facilitate easy use, the approach is also hosted on a publicly available web-server, developed using the R Shiny package (version 1.7.3) [20, 21]. The web tool is further described in the Additional File: Sect. 1.3, and Fig. 2 presents an example of its usage.

### Case studies

Input parameters for included case studies were estimated using publicly available data. Variant frequencies were estimated across people with and without the variant in the familial, ${M}_{F}$, and sporadic, ${M}_{S},$ states in all cases and, in case 1, the unaffected state, ${M}_{U}$. ${M}_{U}$ was integrated into penetrance estimation for case study 1 only to demonstrate the application of the method when sampling from various disease state combinations. This was not applied to other case studies as estimation focusses upon rare variants liable to ascertainment bias in control populations. In all cases, we derived the standard error of these values, ${\sigma }_{\overline{{M }_{X}}}$, to allow for assessment of error in the penetrance estimate. Variant frequency estimates were weighted to calculate $R{\left(X\right)}^{obs}$ among variant-harbouring families from those states modelled using the factors presented in Table 1. Accordingly, $P(F|A)$ and $P(S|A)$ were defined as weighting factors in all cases. $P{\left(A\right)}^{pop}$ is used in all case studies to derive $g$, according to Eqs. (9–11), and is used as a weighting factor in case 1 only.

Sibship size, $N$, was estimated in each case based on the Total Fertility Rates reported in the World Bank database [22] for the world region(s) best representing the sample.

An R script permitting replication of each case study is provided within our GitHub repository (see the ‘Availability of data and materials’ [19]).

#### Case 1: *LRRK2* penetrance for PD

We estimated the penetrance of the *LRRK2* p.Gly2019Ser variant for PD. This case illustrates the flexibility of this method for application using data drawn from several combinations of the defined disease states.

The first-degree familiality rate of PD, about $0.105$, was used to estimate $P(F|A)$ and $P(S|A)$ [23, 24]. $P{\left(A\right)}^{pop}$ was estimated as $1$ in $37$ ($0.027$), the lifetime risk of developing PD [25].

We estimated ${M}_{F.S}$ using data aggregated from 18 European ancestry groups within a sample of $24$ world populations [26]. Of $\mathrm{3,770}$ unrelated people with familial PD manifestations, $126$ (${M}_{F}=0.033, {\sigma }_{\overline{{M }_{X}}}=2.92\times {10}^{-3}$) harboured the *LRRK2* p.Gly2019Ser variant, compared to $130$ of $\mathrm{10,898}$ with sporadic PD (${M}_{S}=0.012, {\sigma }_{\overline{{M }_{S}}}=1.04\times {10}^{-3}$).

As *LRRK2* p.Gly2019Ser occurred in only $2$ members of the unaffected control sample, we estimated ${M}_{U}$ using the larger European (non-Finnish) sample of the gnomAD v2.1.1 (controls) database [27], in which $10$ of $\mathrm{21,383}$ people harboured the variant (${M}_{U}=4.67\times {10}^{-4}, {\sigma }_{\overline{{M }_{U}}}=1.47\times {10}^{-4}$).

We estimated that $g=0.0267$, in accordance with Eqs. (9–11), based on the estimated ${M}_{F,S,U}$, $P(A)$, and $P\left(F|A\right).$

As no single region is representative of the total sample, we estimated that $N=1.572$ by aggregating Total Fertility Rate estimates available in the World Bank database [22] across each of the 18 European populations sampled, weighted by the proportional contribution of each population to the sample (see Additional File 1: Table S1) [26].

Additional region-specific and joint population penetrance estimates for this variant are presented in Additional File 1: Table S2.

#### Case 2: *BMPR2* penetrance for heritable PAH

We estimated the penetrance of variants in the *BMPR2* gene for heritable PAH, a gene for which the low penetrance of pathogenic variants is well established [28].

Input parameters were defined based on only people with idiopathic (sporadic) or heritable PAH diagnoses [17]. This captures people with and without family disease history and excludes PAH manifestations associated with comorbidities or drug exposure.

We estimated $P(F|A)$ and $P\left(S|A\right)$ using the first-degree familiality rate of heritable PAH, about $0.055$ of people affected by either idiopathic or familial PAH [28]. $P{\left(A\right)}^{pop}$ was estimated as $1$ in $20$ ($0.05$), according to an estimated $1$ in $10$ lifetime risk of developing any PAH, and that idiopathic and heritable PAH forms account for approximately $50\%$ of PAH occurrences [28,29].

To minimise any study-specific bias, we applied data from two reports to build independent estimates for each of ${M}_{F,S}$. The first dataset [17], includes $247$ people with familial PAH, of which $202$ harboured *BMPR2* variants (${M}_{F}=0.818, {\sigma }_{\overline{{M }_{F}} }=0.025$), compared to $200$ of $1174$ in the sporadic state (${M}_{S}=0.170, {\sigma }_{\overline{{M }_{S}}}=0.011$). The second dataset [30] identified that $40$ of $58$ people with familial PAH (${M}_{F}=0.690, {\sigma }_{\overline{{M }_{F}} }=0.061$) harboured *BMPR2* variants, compared to $26$ of $126$ in the sporadic state (${M}_{S}=0.206, {\sigma }_{\overline{{M }_{S}}}=0.036$). Variant counts were additionally reported separately for small genetic variations (single nucleotide variants and indels) and structural variants in *BMPR2,* allowing penetrance estimation stratified by variant type. Letting ${M}_{U}=0$, we estimated that $g=0.0401$ for dataset 1, and $g=0.0388$ for dataset 2, in accordance with Eqs. (9–11).

The first dataset may violate two assumptions of our approach: first, information on familial clustering was reportedly unavailable and so some families may be represented more than once in the familial state; second, it is not specified whether disease familiality is defined only by the disease status of first-degree relatives. The second sample overcomes a limitation of the first as each family is represented only once in variant counts. However, it is not reported whether disease states are defined according to the status of first-degree relatives only. As $R{\left(X\right)}^{obs}$ is calculated after weighting ${M}_{F,S}$ by the first-degree familial disease rate, the impact of some bias in variant frequency estimates upon penetrance estimates will be minimised.

The first cohort samples people from Asian, European, and North American populations; French, German and Italian cohorts comprise about 60% of the sample [17]. The second cohort samples people exclusively from Western Europe [30]. We therefore estimated that $N=1.543$ in both instances, the Total Fertility Rate of the European Union in 2018 [22].

#### Cases 3 and 4: *SOD1* and *C9orf72* penetrance for ALS

We estimated the penetrance of variants in the *SOD1* and *C9orf72* genes for ALS. For *SOD1*, we examined the aggregated penetrance of *SOD1* variants harboured by people with ALS. For *C9orf72*, we examined the penetrance of a single pathogenic variant, a hexanucleotide GGGGCC repeat expansion (*C9orf72*^RE^; g.27573529_27573534GGCCCC[30 <]). These penetrances have been historically difficult to establish without incurring kinship-specific biases. They represent ideal candidates for application of our method.

The first-degree familiality rate of ALS, about $0.050$, was applied to define $P(F|A)$ and $P(S|A)$ in these cases [31, 32]. $P{\left(A\right)}^{pop}$ was estimated as $1$ in $400$ (0 $.0025$), the lifetime risk of developing ALS [33].

We drew upon the results of two meta-analyses [34, 35] to estimate ${M}_{F,S}$ for *SOD1* and *C9orf72*^RE^. As variant frequencies differed between Asian and European ancestries, we modelled penetrance separately for each group. We derived ${\sigma }_{\overline{{M }_{F,S}}}$ using z-score conversion from the 95% confidence intervals (95% CIs) reported: for the arbitrary state X,

12 $${\sigma }_{\overline{{M }_{X}} }=\frac{{M}_{X}-{M}_{X}^{95\%lower}}{z}$$

where $z=1.96$ and ${M}_{X}^{95\%lower}$ is the lower 95% CI bound of the estimate ${M}_{X}$.

In Asian ALS populations: *SOD1* variants were harboured by $0.300$ (${\sigma }_{\overline{{M }_{F}} }=0.025$) of people with familial and $0.015$ (${\sigma }_{\overline{{M }_{S}} }=2.55\times {10}^{-3}$) with sporadic disease; *C9orf72*^RE^ was harboured by $0.04$ (${\sigma }_{\overline{{M }_{F}} }=0.010$) of people with familial and $0.01$ (${\sigma }_{\overline{{M }_{S}} }=5.10\times {10}^{-3})$ with sporadic disease. In accordance with Eqs. (9–11), and letting ${M}_{U}=0$, we estimated that $g=0.00243$ for *SOD1*, and $g=0.00247$ for *C9orf72*.

In Europeans, *SOD1* variants were harboured by $0.148$ (${\sigma }_{\overline{{M }_{F}} }=0.017$) of people with familial and $0.012$ (${\sigma }_{\overline{{M }_{S}} }=2.55\times {10}^{-3})$ with sporadic disease; *C9orf72*^RE^ was harboured by $0.32$ (${\sigma }_{\overline{{M }_{F}} }=0.020$) of people with familial and 0.05 (${\sigma }_{\overline{{M }_{S}} }=5.10\times {10}^{-3})$ with sporadic disease. In accordance with Eqs. (9–11), and letting ${M}_{U}=0$, we estimated that $g=0.00245$ for *SOD1*, and $g=0.00234$ for *C9orf72*.

The *SOD1* meta-analysis allowed consideration of the extended kinship when defining familial ALS. The familiality definition used in the *C9orf72* analysis is not stated. As before, the weighting of ${M}_{f,s}$ by the first-degree familial disease rate when calculating $R{\left(X\right)}^{obs}$ will minimise any impact of some bias in variant frequencies upon penetrance estimates.

We tested for equal onset variability (see Additional File 1: Sect. 1.2.2) with the *checkOnsetVariability* R function provided in the associated GitHub repository [19], comparing variability in age of ALS onset for people with *SOD1* or *C9orf72* variants to that of people without variants in these genes. The results (see Additional File 1: Fig. S4) suggested approximately equal onset variability between the *SOD1* and no (*SOD1* or *C9orf72*) variant groups, indicated by visual inspection of the cumulative density plot provided and by an approximately equal time spanned between the first and third quartiles of age of onset across the groups. Onset variability appears more unequal in the *C9orf72* case study, with a ~ 1.36 times shorter interquartile interval for people harbouring *C9orf72*^*RE*^ than the no variant cohort. One of the simulation studies presented in Additional File 1: Sect. 1.2.3; Fig. S11 models a comparable departure from the equal onset variability and demonstrates that a small but tolerable inflation of penetrance estimates may occur if sampling a younger cohort. Since the present penetrance estimates are based on pooled variant frequency estimates from large meta-analyses of variant frequencies in these genes, the present degree of unequal onset variability is unlikely to have impacted penetrance estimation.

In these datasets, the Asian ancestry cohorts were predominantly individuals from East Asia, with small proportion from South Asia. The European ancestry cohorts primarily comprise people from European countries, with some from North America and Australasia. Accordingly, $N$ was estimated for the Asian population samples as $1.823$, the Total Fertility Rate for East Asia and Pacific in 2018, and for the European population as $1.543$, the Total Fertility Rate for the European Union in 2018 [22].

## Results

Here we summarise the input data and results of the case studies modelled (see Table 2). Penetrance estimates are presented both when accounting for residual disease risk $g$ among people with no variant and when assuming that $g=0$; those accounting for $g$ are preferred.

**Table 2:** Penetrance estimation for the present case studies

Case study ^a^	Data subset	Variant frequency in state (standard error)			Average sibship size	Residual disease risk ^a^	States modelled ^c^	Familial disease rate for people with variant across states modelled (95% CI)	Penetrance (95% CI)^e^	
		Familial	Sporadic	Unaffected					Assuming no residual disease risk	Accounting for residual disease risk
-	-	${M}_{F} ({\sigma }_{\overline{{M }_{F}} })$	${M}_{S} ({\sigma }_{\overline{{M }_{S}}})$	${M}_{U}({\sigma }_{\overline{{M }_{U}}})$	$N$	$g$	-	$R(X)$	$f (g=0)$	$f (g=g)$
*LRRK2* p.G2019S for PD [26, 27]	European ancestry	0.033 (2.92 × 10^–3^)	0.012 (1.04 × 10^–3^)	4.67 × 10^–4^ (1.47 × 10^–4^)	1.572^*b.1*^	0.0267	F, S, U	0.113 (0.071, 0.155)	0.37 (0.285, 0.443)	0.334 (0.249, 0.408)
							F, S	0.247 (0.202, 0.292)	0.429 (0.348, 0.509)	0.379 (0.299, 0.461)
							F, U	0.172 (0.081, 0.264)	0.35 (0.247, 0.428)	0.32 (0.215, 0.399)
							S, U	0.388 (0.235, 0.541)^*d*^	0.293 (0.161, 0.45)	0.275 (0.138, 0.438)
*BMPR2* variants for PAH	All variants [17]	0.818 (0.025)	0.170 (0.011)	-	1.543^*b.2*^	0.0401	F, S	0.218 (0.195, 0.242)	0.382 (0.339, 0.426)	0.308 (0.266, 0.351)
	All variants [30]	0.690 (0.061)	0.206 (0.036)	-	﻿1.543^*b.2*^	0.0388	F, S	0.163 (0.111, 0.215)	0.281 (0.186, 0.376)	0.212 (0.12, 0.305)
	SNVs and indels [30]	0.569 (0.065)	0.159 (0.033)	-	1.543^*b.2*^	0.0413	F, S	0.173 (0.107, 0.238)	0.299 (0.179, 0.419)	0.225 (0.11, 0.342)
	Structural variants [30]	0.121 (0.043)	0.048 (0.019)	-	1.543^*b.2*^	0.0475	F, S	0.129 (0.011, 0.246)	0.218 (0.014, 0.432)	0.138 (0, 0.345)
*SOD1* variants for ALS [35]	Asian	0.300 (0.025)	0.015 (2.55 × 10^–3^)	-	1.823^*b.3*^	0.00243	F, S	0.513 (0.420, 0.606)	0.829 (0.665, 1)	0.826 (0.661, 1)
	European	0.148 (0.017)	0.012 (2.55 × 10^–3^)	-	1.543^*b.2*^	0.00245	F, S	0.394 (0.281, 0.506)	0.705 (0.496, 0.933)	0.701 (0.491, 0.926)
*C9orf72*^*RE*^ for ALS [34]	Asian	0.04 (0.010)	0.01 (5.10 × 10^–3^)	-	1.823^*b.3*^	0.00247	F, S	0.174 (0.013, 0.335)	0.263 (0.0156, 0.522)	0.258 (0.0108, 0.518)
	European	0.32 (0.020)	0.05 (5.10 × 10^–3^)	-	1.543^*b.2*^	0.00234	F, S	0.252 (0.208, 0.296)	0.443 (0.363, 0.524)	0.439 (0.358, 0.52)

^a^Disease characteristics of lifetime disease risk ($P{\left(A\right)}_{pop}$) and proportion familial ($P(F|A)$(F|A)) are used as weighting factors (per Table 1) and for calculating $g$ (per Eqs. (9–11), letting $M_{U}=0$ in the ALS and PAH case studies), and are defined as follows: in PD, $P{\left(A\right)}^{pop}=0.027,P\left(F|A\right)=0.105$; in PAH, $P{\left(A\right)}^{pop}=0.05,P\left(F|A\right)=0.055$; in ALS, $P{\left(A\right)}^{pop}=0.0025,P\left(F|A\right)=0.050$. ^b^Estimated using Total Fertility Rates reported for the: populations sampled to calculate variant frequencies (see Additional File 1: Table S1) ^b.1^, European Union^b.2^, or East Asia and Pacific^b.3^ regions in 2018 [22]; ^c^F = familial, S = sporadic, U = unaffected (controls); ^d^Rate of sporadic disease has been calculated here because the familial state is not represented; ^e^Step 4 penetrance estimates are presented, see Additional File 1: Table S5 for unadjusted penetrance estimates derived in Step 3. *PD*, Parkinson’s disease; *PAH*, pulmonary arterial hypertension; *ALS*, amyotrophic lateral sclerosis; *C9orf72*^*RE*^, the pathogenic C9orf72 GGGGCC hexanucleotide repeat expansion; *SNV*, single nucleotide variant; *indel*, small insertions or deletions; *CI*, confidence interval

Estimated penetrance of the *LRRK2* p.Gly2019Ser variant for PD was roughly consistent across the modelled disease state combinations. Additional penetrance estimates across various populations within the dataset from which this European sample was drawn are presented in Additional File 1: Table S2.

The penetrance of *BMPR2* variants for PAH was estimated comparably across the two sample sets, although slightly higher within dataset one [17] than two [30]. Penetrance was also comparable between the defined *BMPR2* variant subtypes of the second sample. Differences in these estimates reflect variation in ${M}_{F,S}$ between the cohorts and may result from a different admix of variants between samples, or unspecified family clustering within the first sample set. It is not known for either dataset whether family history classifications were restricted to first-degree relatives only and so the estimates obtained may be slightly inflated. We note, however, that impact of any inflation was minimised because variant frequency weighting factors were correctly defined. With the available data, these possibilities cannot be explored further.

Penetrance estimates of *SOD1* and *C9orf72* variants for ALS demonstrate consistency within genes across populations and indicate that the penetrance for ALS is greater in people harbouring *SOD1* variants than in those harbouring *C9orf72*^RE^. Additional File 1: Table S3 presents additional penetrance estimates for several widely-described *SOD1* variants: penetrance was estimated as 1 for p.Ala5Val (c.14C > T), 0.644 for p.Ile114Thr (c.341 T > C), and 0 for p.Asp91Ala (c.272A > C). Each estimate made in these case studies may be slightly inflated owing to inclusion of extended kinship within familiality definition. However, as before, accurately defined weighting factors will have minimised this impact.

## Discussion

We have developed a novel approach to estimate the penetrance of genetic variants pathogenic for autosomal dominant traits. The method was tested via simulation studies (see Additional File: Sect. 1.2.3) and application to several case studies.

Our penetrance estimates of the *LRRK2* p.Gly2019Ser variant for PD and of the aggregate penetrance of *BMPR2* variants for PAH closely matched those obtained using other approaches. Previous research on *LRRK2* p.Gly2019Ser estimates its lifetime penetrance between 0.24 (95% CI: 0.135, 0.437) and 0.45 (no CI reported) when analysing data that is not liable to inflation owing to biased selection of familial cases [2]. Longitudinal analysis of disease trends among 53 families harbouring *BMPR2* variants finds penetrance as 0.27 overall, 0.42 for women and 0.14 for men [36]. These case studies additionally demonstrated the importance of considering residual disease risk $g$ for family members not harbouring the variant when estimating penetrance in more common traits. This importance is explored further within simulation studies (see Additional File 1: Sect. 1.2.3; Fig. S8).

The estimates in the *SOD1* and *C9orf72* case studies align with current understanding of penetrance in these ALS genes.

For *SOD1* variants, penetrance for ALS is incomplete and differs between variants [10, 37]. The widely-described p.Ala5Val (formally p.Ala4Val) variant has a recorded penetrance of 0.91 by 70 years of age [38]. Among other variants, penetrance is typically lower [10, 37]. Of those best characterised, p.Ile114Thr approaches complete penetrance in some pedigrees and p.Asp91Ala reaches polymorphic frequency in some populations, with linked ALS presentations typically displaying an autosomal recessive pattern [10, 13, 38]. Our estimates for heterozygous inheritance of these individual variants aligned with these observations (see Additional File 1: Table S3) and highlight the spectrum of penetrance across variants in *SOD1*. Our estimate for the p.Asp91Ala variant in particular supports the hypothesis that it is associated with ALS via a recessive or oligogenic inheritance pattern [39]. The absence of p.Asp91Ala within the familial ALS database sampled further corroborates the finding. Accordingly, our penetrance estimates in Asian and European populations can be taken to suitably represent an aggregated penetrance of risk variants in *SOD1* for ALS; some variation between populations can be expected, reflecting differences in the admix of variants between them.

For *C9orf72*, we modelled the penetrance of its pathogenic hexanucleotide repeat expansion for ALS. Pleiotropy of this variant is widely reported, additionally conferring risk for frontotemporal dementia and, to a lesser degree, other neuropsychiatric conditions [40]. Past penetrance estimates made for this variant are vulnerable to inflation from biased ascertainment of affected people, and the variant is more common among unaffected people than would be expected if these estimates were accurate [12, 40, 41]. A previous study tentatively reports the penetrance of *C9orf72*^*RE*^ for either ALS or frontotemporal dementia as 0.90 by age 83 after attempting to adjust for ascertainment bias within their sample [41]. Accounting for lifetime risk of each phenotype and their respective familiality rates, people of European ancestry harbouring *C9orf72*^RE^ appear to develop ALS or frontotemporal dementia with comparable frequency, we calculated that 1.012 cases of ALS emerge per case of frontotemporal dementia (see Additional File 1: Table S4) [33, 34, 42–44]. It is therefore reasonable to predict that, if the variant has 0.90 penetrance for the joint condition of ALS and frontotemporal dementia, its penetrance of for ALS alone would be around 0.45. The 0.45 estimate is comparable to the upper bound of our findings. However, we note that our calculation does not account for the common co-occurrence of ALS and frontotemporal dementia and that the true penetrance of *C9orf72*^RE^ for the joint ALS-frontotemporal dementia condition is likely lower than the tentative 0.90 estimate.

The method presented has high validity. Internal validity is demonstrated within simulation studies (see Additional File: Sect. 1.2.3). Criterion and face validity are shown across the present case studies, aligning with estimates made using other techniques and current understanding of the assessed cases. Construct validity is also demonstrated: in the ALS case studies, disease risk was greater for those harbouring a pathogenic *SOD1* variant than for those with the *C9orf72* repeat expansion. This aligns with the multi-step model of ALS [45], where harbouring *SOD1* variants is associated with a 2-step disease process, converse to the 3-step process associated with *C9orf72*^RE^.

The data necessary to operate our approach is distinct from other techniques which examine patterns of disease among affected people, allowing assessment of penetrance in unrelated populations rather than families. The estimates are therefore unaffected by kinship-specific modifiers and are instead applicable to the sampled population. Since penetrance may vary according to genetic background, ancestry-specific penetrance estimates are best obtained by stratifying input data according to ancestral groups; this approach is demonstrated in the PD and ALS case studies (see Table 2, Additional File 1: Table S2).

Where analysis is confined to people affected by disease, across the familial and sporadic states, we circumvent the ascertainment biases affecting designs which examine the distribution of a variant between affected and unaffected populations [9]. Where analysis includes data for unaffected samples (i.e. controls harbouring the variant) these would not be avoided; ascertainment of controls compared to cases has equivalent challenges irrespective of the penetrance estimation approach. However, as our method does not require this information if data of familial and sporadic cases are available, this does not majorly limit the approach.

Furthermore, limitations of ascertainment will diminish as huge datasets of genetic and phenotypic information available within public databases become increasingly available. Therefore, the usefulness of penetrance estimates generated through population data will grow alongside the increasing size and scope of genetic data within such datasets [9].

A limitation of this approach is the definition of familiality, which is the occurrence of the studied trait in a first-degree relative under this model. In practice, familial disease may be defined using various criteria, for example considering the disease status of second- or third-degree relatives, or including related diseases that may share a genetic basis [32, 46]. For example, ALS and frontotemporal dementia each share a genetic basis, and considering a family history of frontotemporal dementia is reasonable when assessing familiality in a person with ALS. If the extended kinship is incorporated within familial disease state definitions, then the familial rate will trend upwards and inflate penetrance estimates. Using a wider definition of being affected is acceptable, although it will yield penetrance estimates for the joint condition.

A further caveat is that the model equations assume a particular family structure. It is not feasible to include all possible family configurations for large quantities of summary data however and approximations made are sufficiently close to provide an estimate of penetrance.

This method is suitable for calculating the point, rather than age-dependent, penetrance of pathogenic variants and can be applied to any germline genetic variation associated with a disease via an autosomal dominant inheritance pattern. Penetrance can be derived for an individual variant or for an aggregated set of variants, with the latter indicating an averaged burden of variants meeting the given criteria. We suggest that confidence intervals should be included when using this approach; the size of the interval returned will provide a useful indication of whether the data provided are sufficient for precise penetrance estimation.

When samples include only people harbouring the variant, the method assumes the stability of disease states among sampled families over time. This assumption is typical in case–control research designs, which expect that members of the control sample will not later become cases. However, in traits with age-dependent penetrance, estimates would be influenced by the age at time of sampling. Younger samples would yield reduced estimates if fewer than two family members are affected when sampled and others will only later become affected. A lifetime penetrance estimate would therefore be best obtained within this sampling scenario if sampling people beyond the typical age of onset for the studied disease.

Reasonable lifetime penetrance estimates can however be obtained at earlier sampling times even in circumstances of age-dependent onset when disease state rates are calculated via weighted proportions of variant frequency estimates within those states sampled. This sampling approach was applied in each of the 4 case studies, and follows an assumption that family disease states change comparably over time for people with and without the variant (see Additional File 1: Sect. 1.2.2). Simulation studies demonstrated this relative stability across ages and a relative tolerance to an unequal variability in age of onset profiles between variant and non-variant groups (see Additional File 1: Sect. 1.2.3; Fig. S10-S12).

Point penetrance estimates have several applications, for instance, improving the characterisation of pathogenic variants at a population level, facilitating research involving tested variants and, in particular, aiding clinical trial design by supporting the curation of homogenous study populations. They would have additional utility once gene therapies move towards preventative treatment, giving justification for or against such treatment after accounting for possible side effects and risks.

In a scenario where penetrance can be estimated via multiple approaches, we recommend applying each applicable method, given the complimentary nature of these techniques. If the results of multiple approaches conflict, we would suggest inspection of the suitability of the input data given for each method and to prioritise the result which these best fit.

## Conclusions

Our novel method for penetrance estimation fills an important gap in medical genetics because, utilising population-scale data, it enables the unbiased and valid calculation of penetrance in genetic disease instances that would be otherwise difficult or impossible using existing methods. It serves to expand the range of genetic diseases and variants for which high-quality penetrance estimates can be obtained, as we illustrate in the ALS case studies. Estimates drawn via this approach have clear utility and will be useful for characterisation of pathogenic variants, with benefits for both clinical practice and research. They have wider relevance to the population than those obtained by studying particular kinships and will be more interpretable for clinical professionals.

The tool code is available as an R function on GitHub [19] and the method is available and free to use via a public webserver [20].

## Supplementary Information

**Additional file 1.** This file contains all supplementary methods, figures, and tables referred to throughout this manuscript.

## Data Availability

The datasets generated and/or analysed during the current study are available in our GitHub repository ^19^: https://github.com/ThomasPSpargo/adpenetrance. This includes R scripts used for approach validation as described in this manuscript and Additional File: Sect. 1.2. The datasets supporting the conclusions of this article are included within the article (and its additional file).

## Abbreviations

ALS: Amyotrophic lateral sclerosis
CI: Confidence interval
M_F,S,U,A_: Variant frequencies in the familial, sporadic, unaffected, and affected states
PAH: Pulmonary arterial hypertension
PD: Parkinson’s disease
P(A): Probability of being affected by a given disease
P(F|A): Disease familiality rate
P(S|A): Disease sporadic rate
P(M): Probability of harbouring the variant M
W_F,S,U,A_: Weighting factors for the familial, sporadic, unaffected, and affected states
R(X)^obs^: Observed rate of arbitrary state x
