# Supplementary material for: Calculating variant penetrance from family history of disease and average family size in population-scale data
Source: Genome Med. 2022 Dec 15;14:141. doi: 10.1186/s13073-022-01142-7 (PMC9753373; doi:10.1186/s13073-022-01142-7)
Supplement: Supplementary file 1 — Additional file 1. This file contains all supplementary methods, figures, and tables referred to throughout this manuscript. [file 13073_2022_1142_MOESM1_ESM.pdf]

Calculating variant penetrance from family history of disease and average family size in population-scale data

Supplementary Materials

1. Supplemental Methods
   1.1. Penetrance calculation procedure
   1.2. Approach validation and testing
      1.2.1. Lookup table validation: an alternative maximum-likelihood approach
      1.2.2. Age-dependent penetrance: tolerance to age of sampling
      1.2.3. Simulation studies
   1.3. ADPenetrance: a companion web tool

2. Supplemental Figures
   2.1. Fig S1. Error in unadjusted penetrance estimates across true penetrance values and according to states modelled for a simulated population where sibship sizes follow a given distribution.
   2.2. Fig S2. Errors in unadjusted penetrance estimates across true penetrance values and according to states modelled for a simulated population

Fig S3. Sibship distributions upon which simulated populations were modelled across simulation studies.

   2.3. Fig S4. Cumulative density plots comparing variability in age of ALS onset for people with and without SOD1 or C9orf72 gene variants.
   2.4. Fig S5. Error in penetrance estimates across true penetrance values when $R(X)^{obs}$, N and g are specified correctly in the simulated UK (1974) and Next Steps populations.
   2.5. Fig S6. Error in penetrance estimates according to degree of error in estimation of N.
   2.6. Fig S7. Error in penetrance estimates according to degree of error in estimation of $R(X)^{obs}$.
   2.7. Fig S8. Error in penetrance estimates according to magnitude of disease risk g for people not harbouring the variant.
   2.8. Fig S9. Penetrance according to age of sampling across only families harbouring a variant of lifetime penetrance f.
   2.9. Fig S10. Error in lifetime penetrance estimates according to age of sampling based on variant frequency estimates across a wider disease cohort with equal age of onset variability.
   2.10. Fig S11. Error in lifetime penetrance estimates according to age of sampling based on variant frequency estimates across a wider disease cohort when age of onset density is more compressed among people harbouring the tested variant.
   2.11. Fig S12. Error in lifetime penetrance estimates according to age of sampling based on variant frequency estimates across a wider disease cohort when age of onset density is less compressed among people harbouring the tested variant.

3. Supplemental Tables
   3.1. Table S1 – Sample characteristics and calculation of N for data applied in case study 1
   3.2. Table S2 – Penetrance estimation of the *LRRK2* p.Gly2019Ser variant for Parkinson’s Disease across populations sampled in case study 1
   3.3. Table S3 – Penetrance estimation for widely described *SOD1* variants
   3.4. Table S4 – Estimation of the incidence of amyotrophic lateral sclerosis relative to frontotemporal dementia among people of European ancestry who harbour the pathogenic hexanucleotide GGGGCC repeat expansion of the *C9orf72* gene
   3.5. Table S5 - Comparison of unadjusted penetrance estimates derived for the case studies presented in Table 2 between the lookup table and maximum-likelihood approaches
   3.6. Table S6 - Direction of change in $R(X)^{obs}$ and penetrance estimates according to increases in variant frequency and weighting factor estimates.

4. Supplemental References

1. Supplemental Methods

1.1. Penetrance calculation procedure

Here follows an outline of the present approach to penetrance estimation. This method is available as an R function (R Version 4.1.2) accessible at https://github.com/ThomasPSpargo/adpenetrance/.

Step 1:

To calculate penetrance using this method, we must identify the rate at which one of the defined disease states (familial, sporadic, unaffected, affected) occurs in families harbouring the variant sampled across a valid combination of two or three of these states (see Table 1). This rate is denoted as $R(X)$, and X can be any one of the four disease states for which variant information were provided.

Definitions:
Familial = more than one family member affected
Sporadic = only one family member affected
Unaffected = no family member affected
Affected = at least one family member affected – familial or sporadic not specified.

In Step 1, we determine $R(X)$ as it is observed within input data, $R(X)^{obs}$. If known, $R(X)^{obs}$ can be specified directly, alongside a corresponding indication of the states from which this estimate is derived. If the familial state is represented within input data, then state X is familial. If only the sporadic and unaffected states are represented, then state X is sporadic. If the affected and unaffected states are represented, then state X is affected.

$R(X)^{obs}$ can also be derived as a weighted proportion of heterozygous variant frequency estimates drawn from samples of unrelated people from two or three of the familial, sporadic, and unaffected disease states or the affected and unaffected states. When variant frequency estimates for the familial or sporadic states are included, the frequency of familial, $P(F|A)$, and sporadic, $P(S|A)$, disease among the affected population, A, must feature in weightings; note that, as familial and sporadic states are binary outcomes within the affected population, $P(S|A) = 1 - P(F|A)$. Where the unaffected or affected groups are represented, baseline (e.g. lifetime) risk of a population member being affected, $P(A)^{pop}$, must be included within weightings.

In this weighted proportion calculation, we respectively denote variant frequencies for familial, sporadic, unaffected, and affected states as $M_{F,S,U,A}$, to be weighted by the factors $W_{F,S,U,A}$. Given that representation of any two or three of the familial, sporadic, and unaffected disease states or the affected and unaffected states can be used estimate $R(X)^{obs}$, we let the familial, sporadic, unaffected, and affected states be arbitrarily denoted as the states X, Y, and Z. Accordingly, letting $M_{F,S,U,A}$ and $W_{F,S,U,A}$ arbitrarily be $M_{X,Y,Z}$ and $W_{X,Y,Z}$ for the states X, Y and Z,

$$R(X)^{obs} = \frac{M_X W_X}{M_X W_X + M_Y W_Y} \quad \text{(S1)}$$

if data are given for a valid combination of two disease states, or

$$R(X)^{obs} = \frac{M_X W_X}{M_X W_X + M_Y W_Y + M_Z W_z} \tag{S2}$$

if data are given for the familial, sporadic, and unaffected disease states. Note that all 4 states cannot be specified together as the familial and sporadic states are subsumed within the affected state. For this reason, it is also unsuitable to represent the affected state alongside data for either or both of the familial or sporadic states. Table 1 presents all possible disease state combinations and outlines how the associated weighting factors should be defined to calculate $R(X)^{obs}$.

Step 2:
A lookup table to which $R(X)^{obs}$ can be compared for penetrance estimation is generated here. This table stores a series of $R(X)$ values that would be expected at a given value of penetrance, f_i, in a population with average sibship size N, and (optionally) the residual disease risk g for people who do not harbour the variant, which can be calculated according to equations 9-11 but is assumed to be 0 by default. We denote this series of $R(X)$ values as $R(X)_i^{ex}$. The sibship size N must be defined alongside the data provided for Step 1 and should represent the average sibship size of the sample from which $R(X)^{obs}$ is determined.

$P(familial)$, $P(sporadic)$, and $P(unaffected)$ are first calculated, following equations 5-7, for a sequence of f values, $f_i = (0.0000, 0.0001, \dots, 1.0000)$, at a specified N and g. This produces a series of values for each disease state: $P(familial)_i$, $P(sporadic)_i$, and $P(unaffected)_i$. If the affected state has been specified in Step 1, we calculate the probability of being affected, $P(affected)$, in accordance with equation 8 at each f_i: $P(affected)_i = P(familial)_i + P(sporadic)_i$. $R(X)_i^{ex}$ can then be derived as an unweighted proportion from $P(familial)_i$, $P(sporadic)_i$, $P(unaffected)_i$, and $P(affected)_i$, taking those disease states which were previously represented in Step 1 and ensuring X represents the same state as before. The lookup table is next constructed, storing an index of corresponding $R(X)_i^{ex}$ and f_i values.

Step 3:
The $R(X)^{obs}$ estimate obtained in Step 1 is used to query the lookup table generated in Step 2. The value of $R(X)_i^{ex}$ closest to $R(X)^{obs}$ is identified and the corresponding penetrance value is taken (see Supplemental Methods 1.2.1 and Table S5 for comparison to a maximum-likelihood approach). This value is an uncorrected penetrance estimate, $f^{unadjusted}$, subject to a systematic bias within the approach and should not therefore be taken as the final estimate; this is determined in step 4. Note that $R(X)^{obs} \approx R(X)_i^{ex}$ unless $R(X)^{obs}$ exceeds or is less than the rate of state X expected between $f = 0, \dots, 1$ at N and g.

Step 4:
This step computes the final penetrance estimate to be returned by the method, $f^{adjusted}$. It corrects for systematic bias in the $f^{unadjusted}$ estimate from in Step 3, which diverges from the true penetrance value according to the combination of states modelled, the value of penetrance, and the structure of families sampled (see Fig S1; Fig S2).

In Step 4, firstly, a simulated dataset of 90,000 families is pseudo-randomly generated, where each simulated family is assigned a sibship size of the value $N_i^{(sim)}$. The population generated in this step aims to approximate the sibship structure of the real population sampled for penetrance estimation. To ensure replicability, all pseudo-randomisation in this step is performed using the R seed 24.

By default, simulated sibships follow a Poisson distribution with the lambda defined by the mean sibship size, N, specified for the real sample data. Example simulated Poisson sibship distributions are presented in Fig S2 A.i-D.i. The Poisson distribution was selected as the default simulation distribution as it is a discrete probability distribution useful for estimating the number of events expected to occur within a given time frame. In this instance, an event is having a child (1 sib) and the time frame is the childbearing years for that family. We note that the Poisson distribution assumes the independence of events and that this assumption would not hold in the present instance (i.e. in real populations, the probability of having additional offspring will be influenced by having already had N_i offspring). However, Fig S1 demonstrates that the degree of error made in Step 3 penetrance estimates is comparable between the Poisson distribution (Panel A.ii) and other hypothetical population structures (Panels B.ii-D.ii), including the distribution shown in C.i, which resembles that of a UK 1974 population birth cohort [1]. Therefore, a simulated population in which sib-sizes follow a Poisson distribution can be considered sufficient for approximating the expected error in unadjusted penetrance estimates made using data from randomly sampled populations. This is corroborated by the results of the simulations presented in Supplemental Methods 1.2.3).

If the structure of sibships in the real sample is known, then the user can optionally supply the *adpenetrance* R function with either a vector containing all the sampled sibship sizes or a summary of the sibship distribution, declaring the sibship sizes contained in the sample and the proportion of the sample each sib-size represents. When sibship data are supplied, a simulated sibship distribution is generated based on these data, including only the sibship sizes represented and following its sibship distribution. This 'tailored' simulation population will give more precise $f^{adjusted}$ estimates than those obtained using the default Poisson distribution (see Supplemental Methods 1.2.3). However, the Poisson distribution is sufficiently precise for adjustment when the sibship distribution of the real data are unknown, under the assumption that population sampling is random and does not exclude families of a particular sibship size (e.g. families of sibship size 0 are not excluded).

A sequence of 25 penetrance values between 0.01 and 1 is also defined, representing true penetrance values of a simulated variant, $f^{true\ (sim)}$. For each $f_i^{true\ (sim)}$, equations 5-7 are applied at each of the $N_i^{(sim)}$ sibship sizes within the simulated population to determine the probability of a family of sibship size $N_i^{(sim)}$ being familial, sporadic, or unaffected at that $f_i^{true\ (sim)}$. One of the familial, sporadic, and unaffected states is pseudo-randomly assigned to each simulated family, according to the probabilities expected at their sibship size and the given $f_i^{true\ (sim)}$. An unadjusted Penetrance estimate is then made for the simulated population, $f_i^{unadjusted\ (sim)}$, according to the mean sibship size of the simulated

population $N^{(sim)}$, and the $R(X)$ observed, $R(X)^{obs\,(sim)}$. Here, $N^{(sim)} \approx N$, with small variation between these values reflecting the pseudo-randomisation of population generation, and State X is defined as in Step 1, with $R(X)^{obs\,(sim)}$ being calculated as an unweighted proportion of the probabilities of X across the modelled states within the simulated dataset.

The difference between corresponding values of $f_i^{unadjusted\,(sim)}$ and $f_i^{true\,(sim)}$ is calculated: $f_i^{error\,(sim)} = f_i^{true\,(sim)} - f_i^{unadjusted\,(sim)}$. A positive $f_i^{error\,(sim)}$ indicates underestimation of penetrance, while negative values denote overestimation. The relationship between $f^{error\,(sim)}$ and $f^{unadjusted\,(sim)}$ is then established by fitting an nth degree polynomial regression model, which, by extension, also indicates the relationship between $f^{error\,(sim)}$ and $f^{true\,(sim)}$. Polynomial models between 1 and 5 degrees are tested, and the best fitting model is selected based on the Akaike Information Criterion. Fig S1 (A.ii) and Fig S2 (A.ii-D.ii) display examples of these error curves fitted for simulated populations where sibship sizes follow a Poisson distribution. Dynamic generation of these regression models is necessary to account for required changes in model fit according to population sibship structure (see Fig S2).

The fitted polynomial regression model is then used to predict error in the penetrance estimate made for the real dataset in Step 3 based on the value of $f_i^{unadjusted}$, $f_i^{error\,(predicted)}$. The final penetrance estimate is then determined: $f_i^{adjusted} = f_i^{unadjusted} + f_i^{error\,(predicted)}$. The validity of these penetrance estimates is demonstrated in the simulation studies presented in Supplemental Methods 1.2.3).

Optional step:
Confidence intervals for the penetrance estimate can be derived through the calculus approach to error propagation [2]. For this, standard errors, $\sigma_{\overline{M_{X,Y,Z}}}$, of the variant frequency estimates given in Step 1 are required. Using these errors, we calculate the standard error in of $R(X)^{obs}$, $\sigma_{\overline{R(X)^{obs}}}$:

$$\sigma_{\overline{R(X)^{obs}}} = \sqrt{\left(\frac{\partial R(X)^{obs}}{\partial M_X}\right)^2 \cdot \sigma_{\overline{M_X}}^2 + \left(\frac{\partial R(X)^{obs}}{\partial M_Y}\right)^2 \cdot \sigma_{\overline{M_Y}}^2 + \cdots} \qquad \text{(S3)}$$

Confidence intervals for $R(X)^{obs}$, $\mathrm{CI}_{\mathrm{R(X)}^{obs}}$, can then be obtained through z-score conversion ($CI_{R(X)^{obs}} = R(X)^{obs} \pm z \times \sigma_{\overline{R(X)^{obs}}}$). The lookup table is then queried as in operation 3 for upper and lower bounds of $\mathrm{CI}_{\mathrm{R(X)}^{obs}}$ to attain upper and lower bounds for the $f_i^{unadjusted}$ estimate obtained in Step 3. These values are then adjusted as in Step 4 according to the fitted polynomial regression model, giving the final penetrance estimates at the confidence interval bounds.

1.2. *Approach validation and testing*

The R scripts used for approach validation are available within our GitHub repository: https://github.com/ThomasPSpargo/adpenetrance/.

1.2.1. *Lookup table validation: an alternative maximum-likelihood approach*

The unadjusted penetrance estimates obtained in Step 3, $f^{unadjusted}$, can also be derived following a maximum likelihood approach. To validate the lookup table approach implemented, we additionally derived $f^{unadjusted}$ estimates using Non-Linear Minimisation, leveraging *nlm* and *dbinom* functions available within the R *stats* package (version 4.1.2) [3].

We constructed this validation approach by defining a negative likelihood function which determines, under a binomial distribution, the likelihood of the specified $R(X)^{obs}$ at a given $f^{unadjusted}$ and N. Within this function, values of $R(X)^{obs}$ are transformed into integers so that they represent a number of state X events across a certain number of trials (e.g. the rate 0.394 would be multiplied by three orders of magnitude, giving 394 events across 1000 trials). The probability function is defined using equations 5-7, and according to the states modelled in calculating $R(X)^{obs}$.

Non-Linear Minimisation was then applied to determine the most likely $f^{unadjusted}$ given $R(X)^{obs}$, N, and g. The starting value for minimisation was defined as the $f^{unadjusted}$ estimate previously determined via the Step 3 lookup approach.

This approach was applied to each of the case studies presented in Table 2 and we found negligible difference between the $f^{unadjusted}$ estimates generated within non-linear minimisation and via the lookup table method (see Table S5). Thus, these findings confirm the validity of the lookup table approach. The alternative maximum-likelihood method was not adopted for penetrance calculation to avoid potential issues in model convergence if starting values are not appropriately defined.

1.2.2. *Age-dependent penetrance: tolerance to age of sampling*

The penetrance of variant M for an associated disease is determined within the present method according to $R(X)^{obs}$, N, and g. If age of disease onset varies across people harbouring the variant, then penetrance is also age-dependent. In a sample consisting only of families harbouring variant M, $R(X)^{obs}$ will inherently vary over time as people from sampled families age and become affected. Accordingly, penetrance estimates would be lower at an earlier time of sampling, and not accurately represent the true lifetime penetrance. This effect is demonstrated below within a simulation study (see Supplemental Materials 1.2.3, Fig S9). Accordingly, a lifetime penetrance estimate is best obtained within this scenario when people sampled are beyond the typical age of onset for the studied trait.

Within a second sampling scenario, where $R(X)^{obs}$ is determined indirectly as a weighted proportion of a given disease state across variant frequency estimates (per equations S1 and S2) from samples of people with and without the variant across a valid combination of

disease states, age-dependence will have a smaller effect upon estimation of lifetime penetrance.

This is true if the variability in the rate at which family disease states change over time are comparable between families affected by disease where a variant of interest does and does not occur. To illustrate this assumption with an example: If at a given time 100 of 1000 people with sporadic disease harbour the variant of interest, the variant frequency is 0.1. Suppose then that at a later time of sampling, 200 people of the original sample are now considered 'familial'. If the rate of family disease state change is comparable for people with and without the variant over time, then roughly 180 people without and 20 with the variant would have been reassigned as familial. This leaves 80 of 800 people harbouring the variant in the sporadic sample and the variant frequency remains 0.1. Accordingly, under this assumption, variant frequency estimates within a given disease state will be largely stable over time.

In practice, the rate of change over time is unlikely to correspond exactly between people with and without variant M. However, the assumption is reasonable for a disease with a heritable genetic basis when the tested variant is not thought to be indicative of an entirely distinct onset profile. Accordingly, whether the assumption is true will be influenced by two factors: (1) that variability in age of disease onset is comparable for people who will be affected in their lifetime with and without a given variant, and (2) that the number of disease occurrences (across the range of zero and two or more affected) within families is similar between the groups.

The first of these can be tested by comparing the age of disease onset profile for people with and without a given variant; if the groups have 'equal onset variability' over time, then the assumption is more likely met. The important aspect of this test is that people with and without the variant progress from being unaffected to affected at a similar rate across age; absolute differences in age of onset between group (i.e., where a variant is associated with a younger/older disease phenotype) are tolerated. When equal onset variability is observed, change in $R(X)^{obs}$ over time will be determined by differences number of disease occurrences within families between groups; its estimation will be less affected by age-dependence than when sampling only from families within the variant group.

To facilitate testing of equal onset variability, we have made available an additional R function within the ADPenetrance GitHub repository [4]: *checkOnsetVariability*. This function allows users to supply information regarding age of disease onset for two sample groups (with and without a given variant). The age of onset is then centred for each group by a chosen metric (e.g., mean or median), to enable (base R) plotting of either a density or cumulative density function which overlays onset variability for the two groups. In addition, the function calculates the relative difference in span of time between the first and third quartiles of disease onset in each group. (e.g., if there is an 8-year interval between the first and third quartile for onset among people with variant M, and a 10-year interquartile interval for people without M, then the relative difference is $10/8 = 1.25$, indicating that the variability in disease onset 1.25 is smaller among people with variant M, with less time taken to span the interquartile interval). This number is returned to users of *checkOnsetVariability* as a quantifiable indication of the scale of departure from the equal

onset variability. Values of approximately 1 indicate equal onset variability, values > 1 indicate that the onset interval is shorter for people in the variant group, values < 1 indicate that the onset interval is protracted for people in the variant group. An example of plots returned using the *checkOnsetVariability* function is provided in Fig S4, which presents testing of equal onset variability in the ALS case studies modelled versus a 'no variant' ALS population, characterised by absence of variants in *C9orf72* and *SOD1*.

The relative difference in onset variability returned by *checkOnsetVariability* can be supplied to a further function also available on GitHub [4], *simADPenetrance*, which enables users to perform a simulation study that returns a plot which visualises how much a given degree of departure from the assumption may affect penetrance estimates according to sampling age. The *plyr (*version 1.8.7), *ggplot2* (version 3.4.0) and *reshape2* (version 1.4.4) packages are dependencies for *simADPenetrance* [5-7].

We present figures from simulation studies, performed using the *simADPenetrance* function, which demonstrate accuracy of lifetime penetrance estimation according to age of sampling and degree of departure from the test of equal onset variability. In these simulations, families containing the variant of interest are compared to a wider disease cohort of families without this variant and instead harbouring one of several other variants of varying penetrance. In Fig S10, equal onset variability is observed, while Fig S11 presents a 1.3 relative difference in onset variability, which can be compared to the relative difference of 0.77 (approximately the inverse of 1.3) presented in Fig S12.

The simulations demonstrate reasonable accuracy in penetrance estimation across time of sampling when the assumption is met, and tolerable stability when the assumption violated by the tested degree of departure.

A full description of these simulation studies is provided subsequently (Section 1.2.3), and documentation for *checkOnsetVariability* and *simADPenetrance* is provided on GitHub [4].

1.2.3. Simulation studies

Here we present the results of simulation studies conducted to test the validity of the 4-step approach outlined in Supplemental Methods 1.1. The studies described are split into 2 sets according to the methodology followed for generating simulated families. The simulated datasets used within all studies were generated pseudo-randomly in R with no set seed number and $g = 0$ except where stated.

Across both sets of simulation studies, families were pseudo-randomly generated based on sibship distributions previously reported in two distinct samples (see Fig S3).

The first simulated population (henceforth: the UK population) resembles the sibship distribution across the UK population 1974 birth cohort at the end of their childbearing years (defined as 45 years of age) [1]. The families within this simulated dataset were each pseudo-randomly assigned a sibship size between 0 and 4 according to the probabilities observed in this cohort (see Fig S3) and the mean sibship size, N, is 1.84. The simulation

population was modelled on these data because they describe the most recent birth cohort for which data is available at the completion of childbearing years and because the distribution is representative of a randomly sampled population. The distribution of sibship sizes across this cohort is comparable to other reported UK and USA birth cohorts [1,8].

The second population (henceforth: the NS population) was simulated based on the distribution of sibship sizes reported for the Next Steps dataset, a longitudinal sample of children from England [9]. Simulated families were pseudo-randomly assigned a sibship size between 1 and 7 according to the probabilities observed in the Next Steps sample (see Fig S3) and $N = 3.006$. The simulation cohort was modelled on these data to illustrate the application of the method to a sample not fully representative of the population. In this case, the sample does not include families of sibship size 0.

Set 1:
In the first set of simulation studies, the performance of the method was tested on simulated populations containing 90,000 simulated families.

A series of ground truth penetrance values, f_i^{true}, were generated for testing within each study. For each f_i^{true}, families from the two simulated populations were generated as described above and the familial, sporadic, and unaffected disease state probabilities expected at each of the occurring sibship sizes were calculated using equations 5-7. One of these three disease states was then pseudo-randomly assigned to each family with the probabilities expected in a family of that the sibship size. Penetrance estimates, $f_i^{adjusted}$, were then made for the population simulated under the specifications of that study. $f_i^{adjusted}$ estimates were made for each possible disease state combination, producing five $f_i^{adjusted}$ estimates for each value of f_i^{true}, across the combinations of states modelled. The error in $f_i^{adjusted}$ was then determined: $f_i^{error} = f_i^{adjusted} - f_i^{true}$. Positive f_i^{error} values indicate overestimation of penetrance, while negative values indicate underestimation.

In each study, to test the two estimate adjustment approaches allowed in Step 4, we estimated f_i^{error} firstly when the method is supplied no information about the distribution of sibship sizes in the sample data and secondly when this information is supplied. As described in Step 4 (see Supplemental Methods 1.1), the former condition adjusts $f_i^{unadjusted}$ by predicted error in the estimate under a polynomial regression model fitted to a population simulated within the method in which sibships follow a Poisson distribution. The latter condition 'tailors' adjustment of $f_i^{unadjusted}$, by fitting the regression model to a population simulated within the method which directly approximates the real sample data.

Validation under correct parameter specification

We first tested the approach by examining the accuracy of penetrance estimates made using correctly specified input parameters in simulated UK and NS populations harbouring hypothetical variants with known true penetrance values. A sequence of 20 ground truth penetrance values was first defined: $f_i^{true} = (0.05, 0.10, \ldots, 1)$ and the populations were simulated as described above. To examine the influence of g, we simulated scenarios where

$g = (0,0.001,0.1)$. Penetrance estimates, $f_i^{adjusted}$, were made for these populations, defining N according to the mean sibship size of that sample, approximately 1.84 for the UK and 3.01 for the NS populations, and with $R(X)^{obs}$ calculated across all possible disease state combinations. f_i^{error} was then determined. This simulation was repeated 5 times for each value of f_i^{true}, and the results are shown in Fig S5, averaged across repetitions to determine the mean f_i^{error} observed at each value of f_i^{true}, across each of the disease state combinations. These findings evidence the validity and accuracy of penetrance estimates generated via this approach. They also demonstrate the benefit of supplying about the distribution of sibships in the sample data when this is known; this benefit is greater if sample data does not accurately represent sibship sizes across the population (e.g., where the NS dataset contains no families of sibship size 0).

Simulation under incorrect parameter specification

Misspecification of sibship size:
This simulation study examines the accuracy of penetrance estimates when the mean sibship size of sample populations is incorrectly defined. We simulate a wide range of misspecification for sibship size here, although it is likely that degree of misspecification in N would be relatively small for any population-representative sample.

Several values of true penetrance were defined: $f_i^{true} = (0.10,0.25,0.50,0.75,1.00)$. A sequence of values to represent the degree of misspecification in mean sibship size was also specified: $N_i^{modify} = (-1.5, -1.0, \ldots, 3.0)$. The simulated UK and NS populations were generated as before and estimates of $f_i^{adjusted}$ were made, calculating $R(X)^{obs}$ across all possible disease state combinations and defining N according to the mean sibship size of that sample, approximately 1.84 for the UK and 3.01 for the NS populations, adjusted by each value of N_i^{modify}. For instance, if $N = 1.84$ and $N_i^{modify} = -1.5$, penetrance would be estimated based on $N = 0.34$. f_i^{error} was then determined. This simulation was repeated 3 times for each value of f_i^{true}, and the results were averaged across these repetitions.

The results of these simulations are presented in Fig S6. The increased impact of misspecifying N upon penetrance estimates in the UK compared to NS populations reflects that the difference in disease state rates between a family of 0 sibs compared to a family of 1 sibs is greater than between 1 and 2 or 2 and 3 sib families (etc.); this difference is illustrated in the original description of this disease model [10]. Accordingly, misspecified, and particularly underestimated, N will be more impactful on penetrance estimation in the UK population, which has a lower mean sibship size than NS, since variation in disease state rates is greater between individual family sizes when there are fewer sibs.

Misspecification of disease state rates:
This simulation study examines the accuracy of penetrance estimates when $R(X)^{obs}$ is incorrectly estimated. $R(X)^{obs}$ can be supplied directly to the tool or estimated from variant frequency estimates and weighting factors when supplying any valid disease state combination (see Table 1). Estimates of $R(X)^{obs}$, and subsequently penetrance, increase alongside increases in M_X or W_X, and decrease alongside increases $M_{Y,Z}$ or $W_{Y,Z}$. Table S6

summarises the direction of change in $R(X)^{obs}$ and associated penetrance estimates when values of each input parameter increase for each of the valid disease state combinations.

In this simulation study, several values of true penetrance were defined: $f_i^{true} = (0.10, 0.25, 0.50, 0.75, 1.00)$. A sequence of values to represent the degree of error in disease state rate estimates was also specified: $R(X)_i^{modify} = (-0.15, -0.10 \dots, 0.15)$. The UK and NS populations were simulated as before. $R(X)^{obs}$ was calculated for a given f_i^{true} across each of the five possible disease combinations, with the $R(X)^{obs}$ value to be defined in penetrance estimation being adjusted across each value of $R(X)_i^{modify}$; any adjusted $R(X)^{obs}$ values falling outside of the 0 to 1 interval were truncated to be 1e-10 if below that interval or 1 if above. Penetrance estimates, $f_i^{adjusted}$, were made for the simulated UK and NS populations, defining N according to the mean sibship size of that sample, approximately 1.84 for the UK and 3.01 for the NS populations, and $R(X)^{obs}$ by the adjusted value obtained. For instance, if $R(X)^{obs} = 0.366$ and $R(X)_i^{modify} = 0.15$, then penetrance would be estimated based on $R(X)^{obs} = 0.516$. f_i^{error} was then determined for all estimates made. This simulation was repeated 3 times for each value of f_i^{true}, averaging the results across these repetitions. The results of this simulation study are presented in Fig S7.

Simulation to test influence of g accuracy upon estimate accuracy

Here we examine how the importance of specifying residual disease risk g varies for penetrance estimation according to the prevalence of the disease, reflected in increased g. We estimate penetrance when g is correctly specified and when assumed that $g = 0$. This is tested for a series of values, where $g = (0, 0.001, 0.025, 0.050, 0.75, \dots, 0.2)$. Several values of true penetrance were defined: $f_i^{true} = (0.25, 0.50, 0.75, 1.00)$. Penetrance estimates, $f_i^{adjusted}$, were made for the UK and NS populations, defining N according to the mean sibship size of each simulated sample, approximately 1.84 for UK and 3.01 for NS, and with $R(X)^{obs}$ calculated across all possible disease state combinations. f_i^{error} was then determined. This simulation was repeated 3 times for each value of f_i^{true}, and the results were averaged across each repetition.

The results of this simulation study are shown in Fig S8. It illustrates that when the disease is rare in the population, and therefore g is small, accounting for g is less critical for attaining accurate penetrance estimates. However, for more common diseases, this is essential.

Set 2:
This second set of simulation studies simulations aims to test the influence of age sampling upon the accuracy of penetrance estimation in phenotypes with age-dependent onset. Several simulation scenarios are presented.

In each simulation, several values of true penetrance were tested: $f_i^{true} = (0.25, 0.50, 0.75, 1.00)$. Each simulation was repeated 3 times for each value of f_i^{true}, and the results were averaged across each repetition. As above, penetrance estimates, $f_i^{adjusted}$, were made for simulated representations of the UK and NS populations, defining N according to the mean sibship size of each simulated sample, approximately 1.84 for UK and 3.01 for NS, and with $R(X)^{obs}$ calculated across all possible disease state combinations.

f_i^{error}, which in this simulation reflects difference between the estimate and lifetime penetrance at each time of sampling, was then determined. Each simulation was repeated 3 times for each value of f_i^{true} and the results were averaged across these triplicates.

As before, population structures were firstly generated by pseudo-randomly assigning each family a given sibship size, between 0 and 4 for the UK population and 1 and 7 for the NS sample according to the probabilities of each sibship size per population (see Fig S3).

For a given family of sibship size N_i, individual family members are then generated, consisting of two parents and N_i siblings. Family members are each assigned relative ages at the time of first sampling, where 0 indicates the final age before the simulated disease becomes onsets in any person with or without the variant. The youngest of N_i siblings is assigned age 0, and the other siblings are, using the rnorm function, pseudo-randomly assigned age differences of mean 3 (SD=0.75) which are then summed relative to the age of the next youngest sibling and rounded to the nearest integer. This produces N_i siblings separated by ~3 years of age. Each of the two parental ages are also assigned using rnorm. In a family with $N_i = 0 \; or \; 1$, 'parental' ages are generated as mean age 25 (SD=3), rounded to the nearest integer. If $N_i > 1$, the mean age is adjusted in line with the age of the oldest sibling (e.g., if the oldest sibling is 9, then mean parental age is 34).

We simulate a disease which may onset across a 10-year period, where (as above) 0 represents the final age before disease could onset and 10 represents age by which all disease occurrences have onset. We optionally allow the onset window to scale separately within this 10-year window according to variant status (whether or not the variant with penetrance f_i^{true} is harboured). To give an example scenario: all disease occurrences will onset between ages 1 and 10, but onset for people with a variant of f_i^{true} onset may be from ages 1 to 7 versus 1 to 10 in people not harbouring f_i^{true}. Letting the onset scale to be distinct according to variant status enabled us to test the impact of deviation from equal onset variability (see Supplementary Methods 1.2.2). Except where specified, these simulations let disease risk scale equally and onset between times 1 and 10 for people with and without f_i^{true}.

Accordingly, age-dependent disease risk is defined as a proportion of the lifetime risk to an individual according to their current age relative to the disease onset period and whether they harbour, do not harbour, or have 50% probability of inheriting the variant M which has lifetime penetrance f. Accordingly, the disease probability, $P(A)$, for an individual at relative age j is:

$$P(A)_j^M = Q_j^f \times f \, , \qquad (S4)$$

if they harbour M, and Q_j^f is the proportion of people with the variant of lifetime penetrance f affected by time point j; Then,

$$P(A)_j^{M'} = Q_j^g \times g \, , \qquad (S5)$$

if variant M is absent, denoted M', where Q_j^g is the proportion of people with residual risk g who are affected by time point j; Finally,

$$P(A)_j^{M^{0.5}} = \frac{Q_j^f \times f}{2} + \frac{Q_j^g \times g}{2} \text{ ,} \qquad (S6)$$

if they have 0.5 probability of inheriting M from a variant-harbouring parent, denoted $M^{0.5}$. Equations S4-S6 mirror equations 2-4 of the main manuscript, with the integration of the Q term.

Let $t = (0, \dots, t, \dots, T)$ denote the time from the first sampling (at $t = 0$) until and including the time when the youngest family member reaches the final age for disease to onset, T. We simulate, using the rbinom function, whether each family member is affected at age j_t, according to the probability relevant to that person based on their variant status ($M, M', or\ M^{0.5}$) per S4-S6. We then sum the number of affected family members at each t, and define the family as 'unaffected' if no family member has disease at t, 'sporadic' if one family member has disease, or 'familial' if two or more family members have disease.

Families generated across the simulated population are then combined. When the number of sampling points until T varies between families, disease state assignments at $t = T$ are duplicated for those families with fewer sampling points until length of t is equal across the population. Penetrance is then estimated for each of the 5 possible disease state combinations at each time t.

Several simulation studies are now presented, demonstrate the effect of age across several scenarios.

Age-dependence when sampling only families harbouring interest variant.

As described in Supplemental Methods 1.2.2, $R(X)^{obs}$ will vary greatly in traits with age-dependent onset according to age of sampling when calculated directly from the observed proportions of disease states across a cohort consisting only of people harbouring the variant. We simulate this scenario by generating a cohort of 100,000 families per the above method where each family contains one variant-harbouring parent, one parent not harbouring the variant, and N_i siblings who have a 50% chance of inheriting the variant. We estimate penetrance based on disease state proportions across the sample for each of the 5 possible disease state combinations at each of $t = 0, \dots, T$ representing the period across which the youngest sibling of each family could become affected.

Fig S9 presents the results of this simulation. Penetrance estimates varied most when sampling includes the Familial state since most Familial state occurrences will emerge across this time period. Sampling the Sporadic or Affected relative to the Unaffected states has smaller degree of change since the elder generation already have the maximum lifetime risk of disease by $t = 0$. Should $R(X)^{obs}$ be estimated based on disease state proportions across a sample of only people harbouring the variant, we suggest that lifetime penetrance is best estimated based on people in the sample who have passed a typical age for disease onset and since family disease states can reasonably be expected not to change further.

Age-dependence when sampling across families with or without variant across a disease cohort

Age-dependence will affect lifetime penetrance estimation less substantially when $R(X)^{obs}$ is estimated from variant frequencies within each disease state and weighting factors defined by the general characteristics of the disease (see Table 1).

We simulate this scenario by generating a general disease cohort across which only certain families harbour the variant of interest, M, which has lifetime penetrance f_i^{true}. Variant M occurs within 100,000 of the generated families. A further 100,000 families are generated, where no family member harbours M, and instead occurs one of several other variants with autosomal dominant inheritance for the disease. Disease risks per age associated with these competing variants are generated as per equations S4-S6, but for further variants with lifetime penetrance $f_i^{competing} = (0.2,0.4,0.6,0.8,1.0)$; 20,000 families are generated for each of the 5 competing variants.

Accordingly, we simulated a total of 200,000 families. Each family contains one parent harbouring variant M or one of the variants with $f_i^{competing}$, one parent not harbouring the variant of that family, and N_i siblings who have a 50% chance of inheriting the variant. We estimate penetrance, across each of the 5 possible disease state combinations for times $t = 0, \ldots, T$ representing the time across which the youngest sibling of each family could become affected. $R(X)^{obs}$ is calculated in accordance with Table 1 and Equations S1 and S2 as a weighted proportion of the relevant variant frequency estimates observed at each t and the appropriate weighting factors. At all times, weighting factors were defined according to their value at the final sampling time ($t = T$).

Fig S10 displays the results of this simulation. After Step-4 error correction, and for $f_i = (0.25,0.5,0,75)$ penetrance estimated diverged from the true penetrance by no more than 5% at most sampling times and disease state combinations. When $f_i = 1.0$, error was somewhat greater when sampling the familial, sporadic and unaffected, or the familial and sporadic states, but within a tolerable distance of true penetrance across all times of sampling. For all values of f_i penetrance was more accurately estimated as age approached the maximum lifetime risk.

In two further simulations, we modelled scenarios alike the previous simulation, but with unequal onset variability between groups. Thus, the onset window for disease differed among people with variant M and those with the competing variants (For example of this, see Fig S4). In the first simulation, we let the onset window for people with the variant be 1.3 times shorter than for those without the variant (This is comparable to the relative difference in time spanned by the interquartile interval in people with ALS harbouring the *C9orf72* variant compared to people with no *C9orf72* or *SOD1* variant; shown in Fig S4). Accordingly, in this simulation all families in which variant M occurred reached their final disease state assignment by $t = 8$, as opposed to $t = 10$ for families where a competitor variant occurred. The results of this simulation are presented in Fig S11. In the second simulation, we test the inverse of the previous analysis, with the relative onset variability of 0.77 (≈1/1.3), letting instead the onset window be shorter for people harbouring competitor variants (reaching final family disease states by $t = 8$). The results of this simulation are given in Fig S12.

In both simulations where the variability of disease onset differed between people with and without the variant, penetrance was estimated with tolerable accuracy across all ages and values of f_i^{true}. However, further departure from equal onset variability would have greater impact upon penetrance estimation (see Supplementary Methods 1.2.2).

1.3. ***ADPenetrance: a companion web tool***

This method of penetrance calculation is additionally available as an open-access web tool accessible at https://adpenetrance.rosalind.kcl.ac.uk. This was coded in R (Version 4.1.2) and leverages the R Shiny package (Version 1.7.3) [11]. An example of the interface and output of this tool is shown in Figure 2, as applied to estimation of *SOD1* variant penetrance for ALS using data from a European sample as described in case study 3.

This tool can be used calculate penetrance for a given variant based on an estimate of $R(X)^{obs}$, a defined sibship size, and an estimate of g. State X is assigned to a particular state based on which disease states are included within input data, as indicated by the user. Those states represented can be any two or all three of the familial, sporadic, and unaffected states or the unaffected and affected states. If the familial state is represented within input data, then state X is familial. If only the sporadic and unaffected states are represented, then state X is sporadic. If the affected and unaffected states are represented, then state X is affected.

The user can derive $R(X)^{obs}$ independently, manually specifying the rate of the state requested by the tool. Alternatively, they can provide variant characteristics and weighting factors (see Table 1), in order to calculate $R(X)^{obs}$ as described in Step 1. These variant characteristics can be given in each disease state as either (1) variant counts and sample size among population-based samples or (2) directly as variant frequencies.

If data are given using variant counts and sample sizes for each disease state, then the error propagation step is included by default, deriving the standard error for each variant frequency from these values. If data are given using variant frequencies or if $R(X)^{obs}$ is provided directly, then the user can opt to provide error terms for those estimates specified to enable error propagation. Error terms can be given either as standard errors or as confidence intervals from which standard errors are derived via z-score conversion. The user is asked to select which of these will be provided and, where confidence intervals are given, should indicate the level of confidence that these represent (95% confidence is assumed by default). Wherever error propagation is performed, the user will also need to specify the desired confidence level for the penetrance estimate output. This is to be selected from a series of options, where z-score conversion is used to transform the standard error of $R(X)^{obs}$ into the upper and lower confidence interval bounds of this estimate, which can then be used to estimate the bounds of the penetrance estimate.

The user must also indicate the average sibship size, N across the sample set. This can be specified either manually or by querying a repository of Total Fertility Rate estimates across many world regions which we have integrated within the tool [12].

g is assumed to equal 0 by default and can optionally be specified to indicate residual disease risk for people within sampled families who do not harbour the tested variant. This

term is important for more common phenotypes (e.g., where $g > 0.01$) but will have less influence upon penetrance estimation when the $g \approx 0$, as would be the case for rare traits.

Once input data are specified, the tool can be operated and $R(X)_i^{ex}$ is calculated for all values of f_i between 0 and 1 at increasing increments of 0.0001. Penetrance is then estimated as in Steps 3 and 4 and a results table is produced.

The results Table presents $R(X)^{obs}$ and the estimated $R(X)_i^{ex}$, $f_i^{unadjusted}$, and $f_i^{adjusted}$ values to which this corresponds, additionally noting which state X represents. $f^{adjusted}$ should be taken as the penetrance estimate. If error propagation is performed, upper and lower confidence intervals and the standard error of the $R(X)^{obs}$ will be provided, alongside corresponding confidence intervals for $R(X)^{ex}$, $f^{unadjusted}$, and $f^{adjusted}$.

2. Supplemental Figures

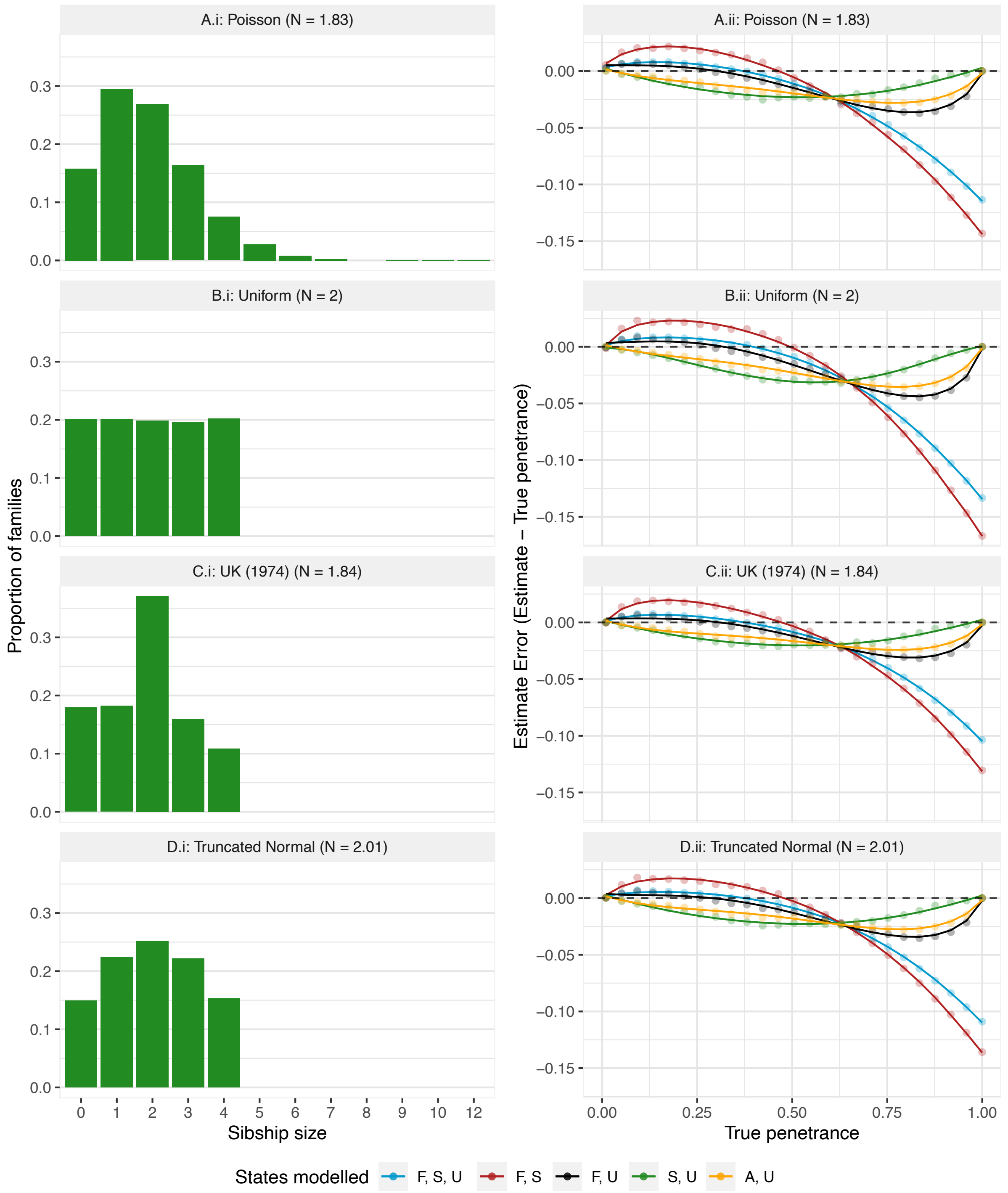

Fig S1. Error in unadjusted penetrance estimates across true penetrance values and according to states modelled for a simulated population where sibship sizes follow a given distribution.

Note: N = mean sibship size, F = familial, S = sporadic, U = unaffected, A = affected. Panels A.i-D.i show the distribution of sibship sizes across simulated families. Panels A.ii-D.ii display errors in penetrance estimates associated with the corresponding population structure - zero indicates a perfect penetrance estimate, positive values indicate overestimation and negative values underestimation; plotted points display raw error values calculated at each true penetrance value and plotted lines display error values predicted under a fitted polynomial regression model.

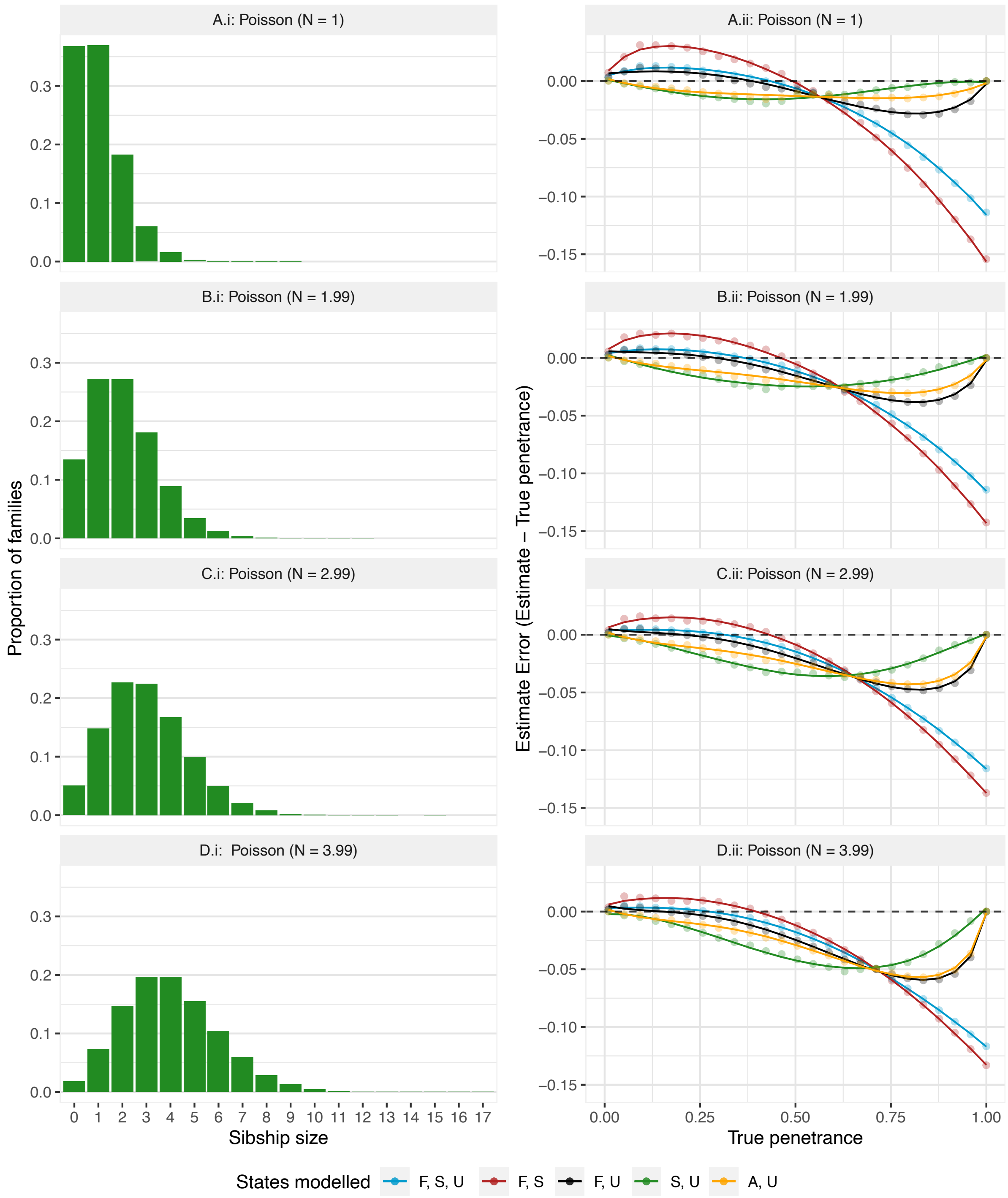

Fig S2. Errors in unadjusted penetrance estimates across true penetrance values and according to states modelled for a simulated population.

Note: Sibship sizes in the simulated data follow a Poisson distribution varying by mean sibship size (lambda). N = mean sibship size, F = familial, S = sporadic, U = unaffected, A = affected. Panels A.i-D.i show the distribution of sibship sizes across simulated families. Panels A.ii-D.ii display errors in penetrance estimates associated with each corresponding population structure - zero indicates a perfect penetrance estimate, positive values indicate overestimation and negative values underestimation; plotted points display raw error values calculated at each true penetrance value and plotted lines display error values predicted under a fitted polynomial regression model.

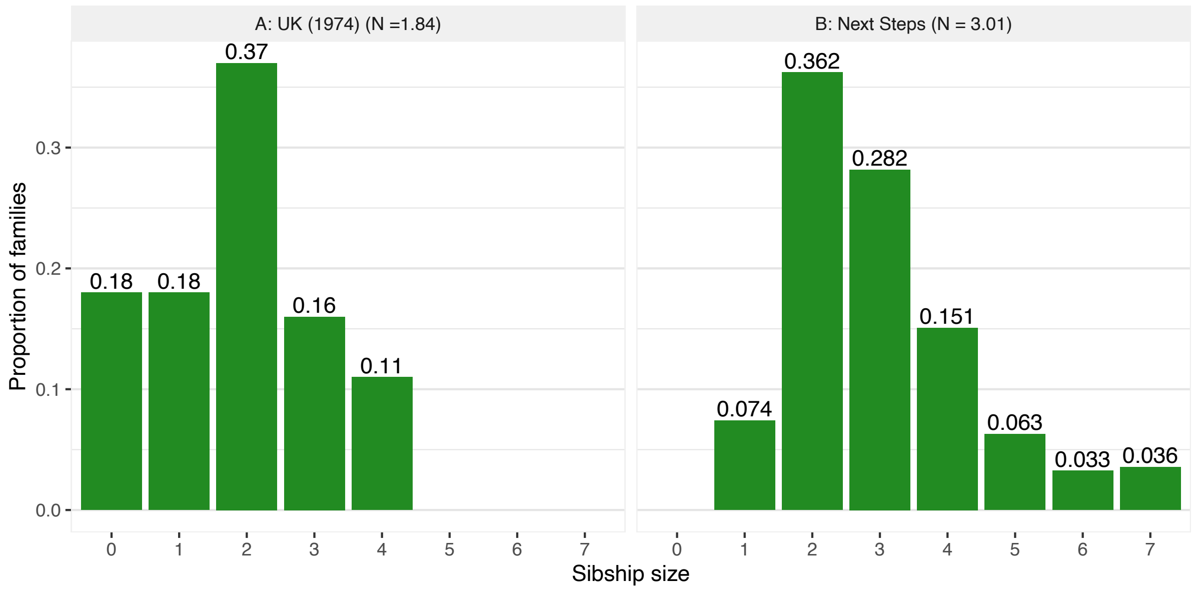

Fig S3. Sibship distributions upon which simulated populations were modelled across simulation studies.

Note: N = mean sibship size. Panel A presents the sibship distribution for the UK population 1974 birth cohort at the completion of their childbearing years; note that the original data reports sibships above size 4 within a collapsed '4 or more' category [1]. Panel B presents the sibship distribution across English families sampled in the Next Steps cohort study; note that the original data reports sibships above size 7 within a collapsed '7 or more' category [9].

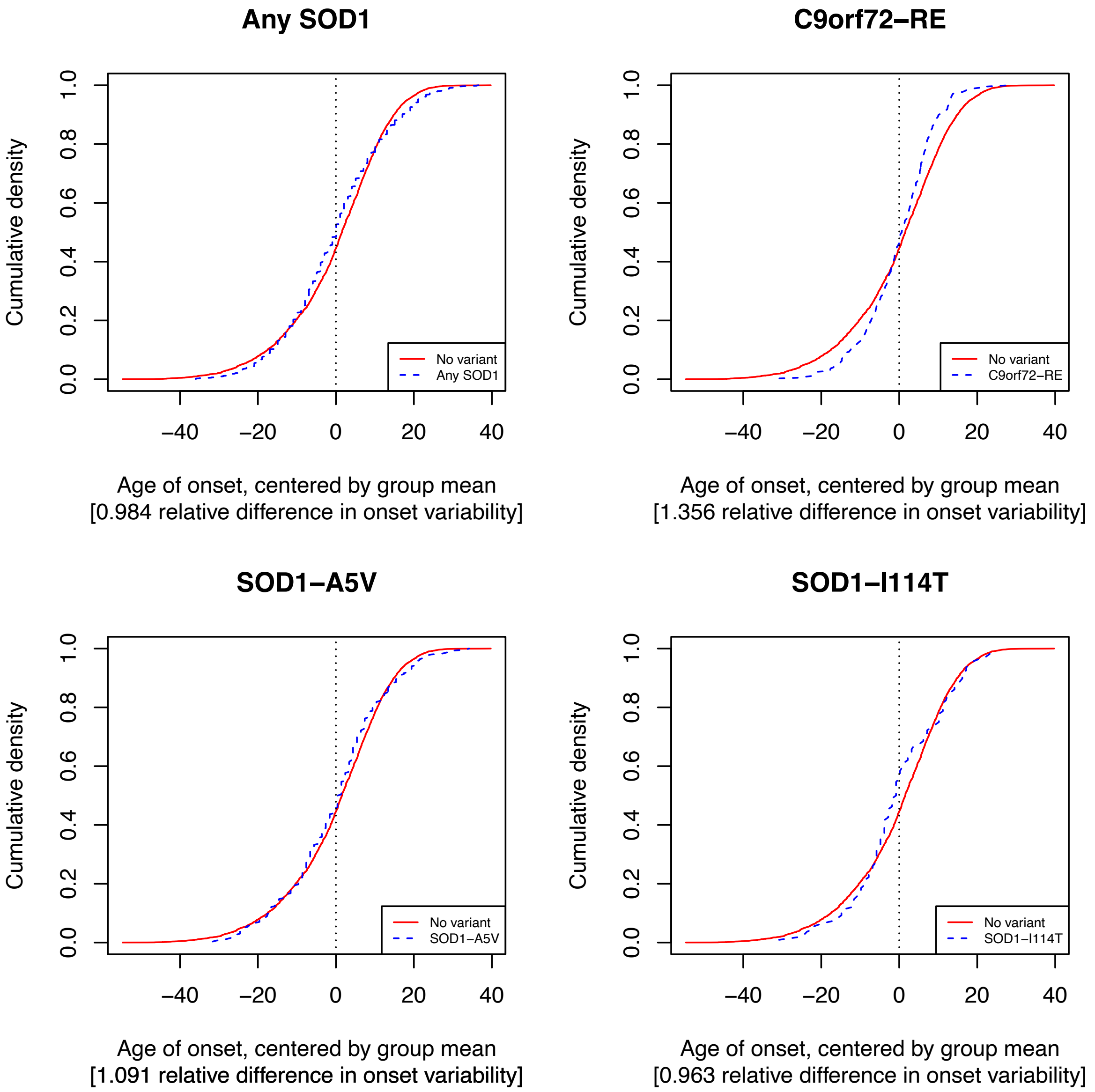

Fig S4. Cumulative density plots comparing variability in age of ALS onset for people with and without SOD1 or C9orf72 gene variants.

Note: Onset distributions for the No variant (n = 5568) and C9orf72-RE (n = 353) groups are derived from people with ALS from Project MinE [13,14]. Those for Any SOD1 (n = 1315), SOD1-A5V (n = 298), and SOD1-I114T (n = 108) are from a multicentre cohort of people with SOD1 variants [15]. The indicated relative difference in onset variability indicates quantifies the relative difference in time between the first and third quartile of disease onset for the 'No variant' vs variant groups; values equal to 1 indicate the similar variability age of onset between groups, >1 indicate a shorter interquartile interval in the variant group, while <1 indicates longer interval for the variant group.

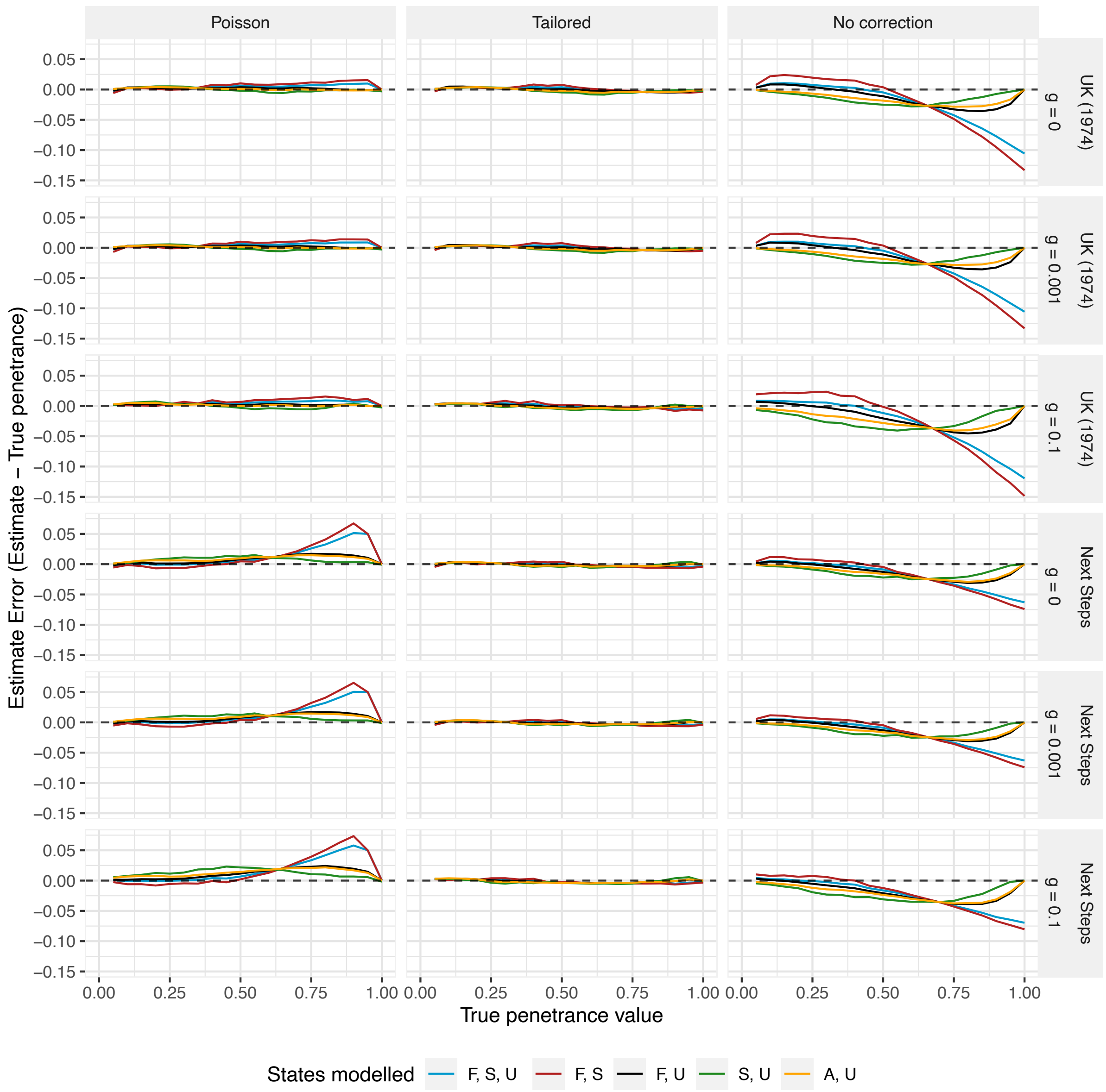

Fig S5. Error in penetrance estimates across true penetrance values when $R(X)^{obs}$, N and g are specified correctly in the simulated UK (1974) and Next Steps populations.

Note: Zero indicates a perfect penetrance estimate, positive values indicate overestimation and negative values underestimation. Plot lines represent estimates made when $R(X)^{obs}$ is defined according to different disease state combinations; F = familial, S = sporadic, U = unaffected, and A = affected - state X is the first state named. The panel rows stratify firstly by population simulated (see Fig S3) and second by the indicated value of residual disease risk g for people not harbouring the tested variant. The columns stratify by Step-4 estimate adjustment approach (see Supplemental Methods 1.1), which follows either the default approach (denoted Poisson), is tailored to errors predicted under an internally-simulated sibship distribution directly approximating the sample data (denoted Tailored), or displays error with no adjustment made to penetrance estimates (denoted No correction).

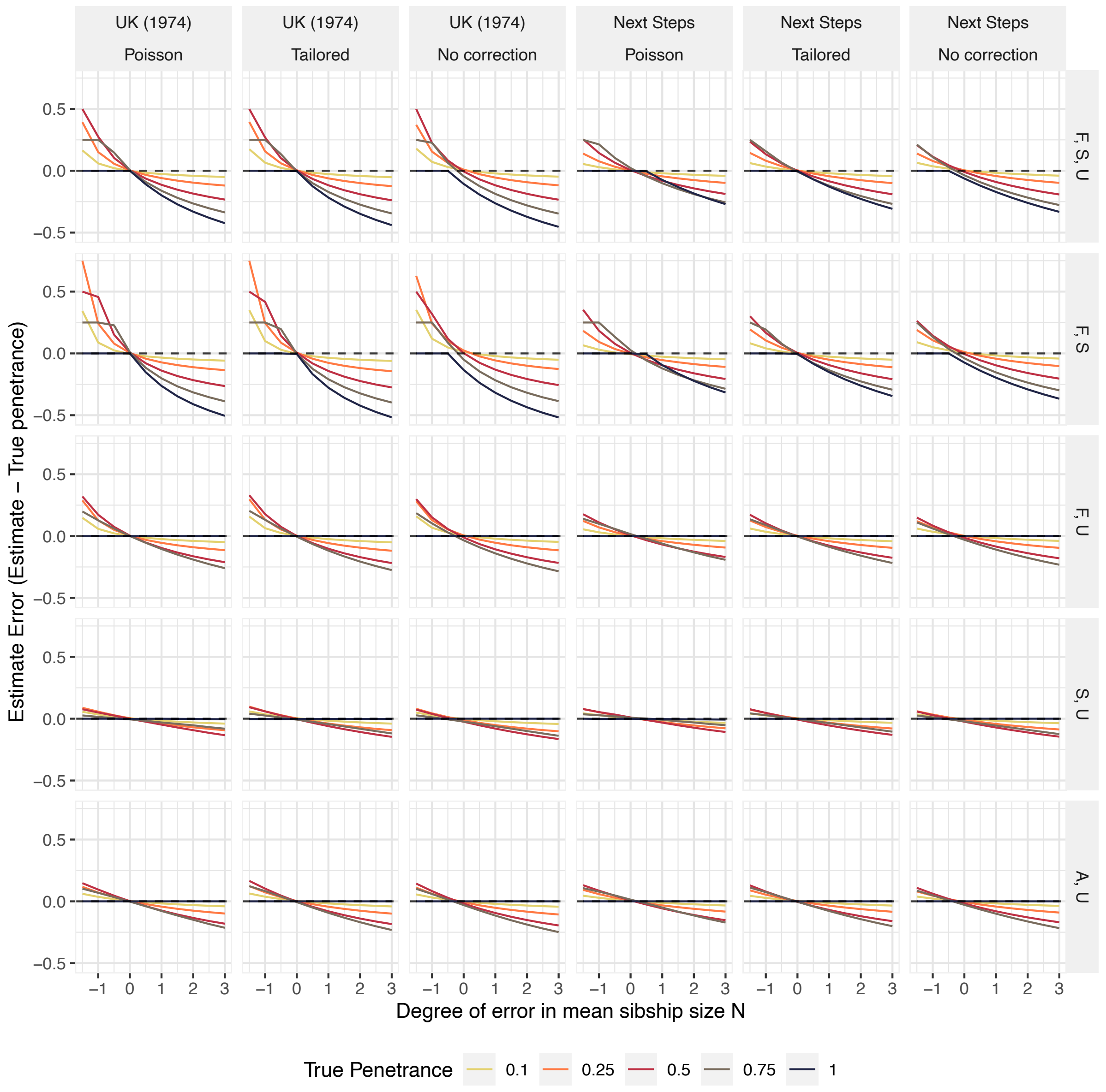

Fig S6. Error in penetrance estimates according to degree of error in estimation of N.

Note: Zero indicates a perfect penetrance estimate, positive values indicate overestimation and negative values underestimation. Plot lines represent different true penetrance values. Panel columns stratify by population simulated (see Fig S3) and by Step-4 estimate adjustment approach (see Supplemental Methods 1.1), which follows either the default approach (denoted Poisson), is tailored to errors predicted under an internally-simulated sibship distribution directly approximating the sample data (denoted Tailored), or displays error with no adjustment made to penetrance estimates (denoted No correction). Panel rows stratify estimates according to the disease state combination from which $R(X)^{obs}$ is defined; F = familial, S = sporadic, U = unaffected, and A = affected - state X is the first state named.

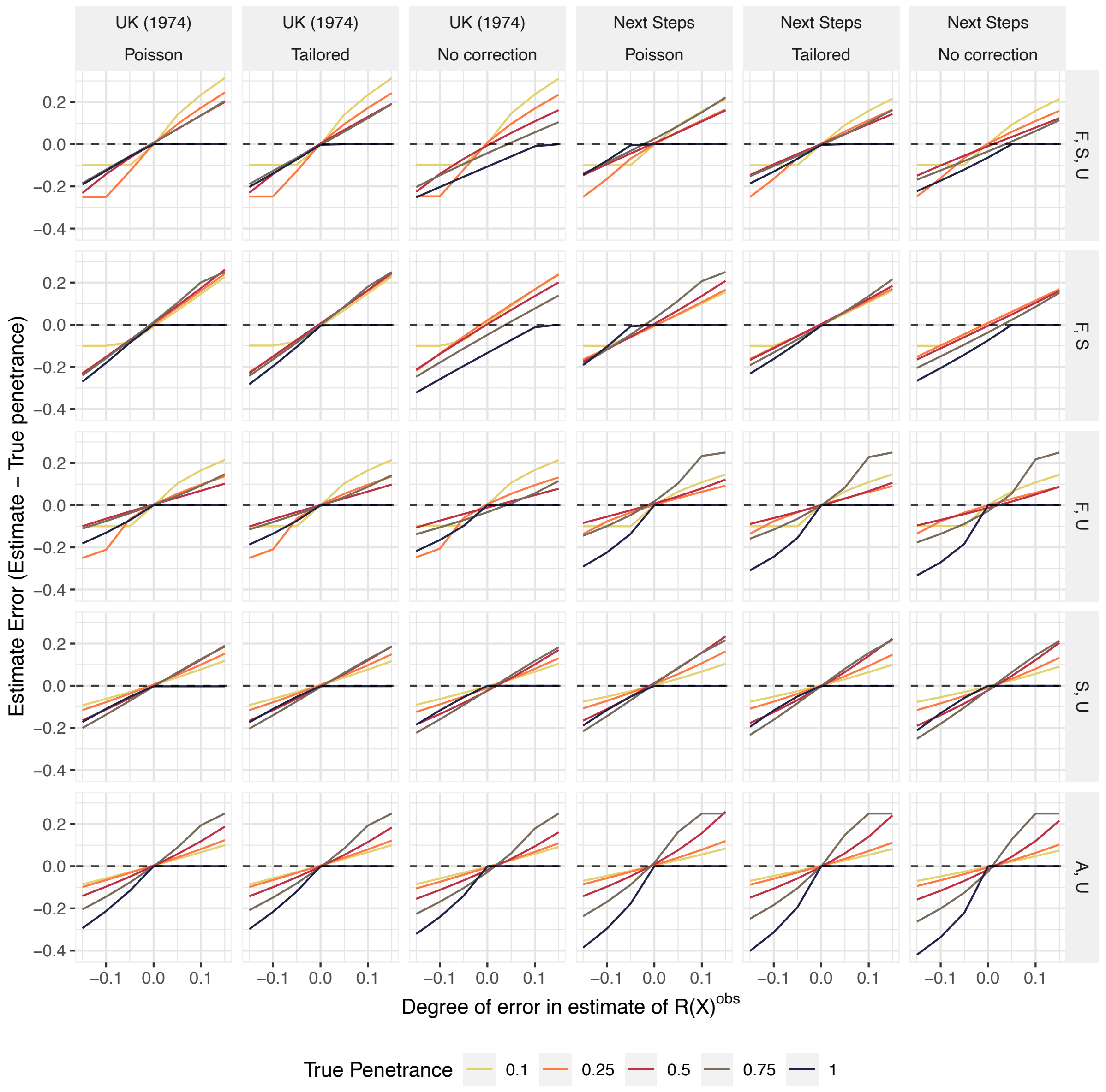

Fig S7. Error in penetrance estimates according to degree of error in estimation of $R(X)^{obs}$.

Note: Zero indicates a perfect penetrance estimate, positive values indicate overestimation and negative values underestimation. Plot lines represent different true penetrance values. Panel columns stratify by population simulated (see Fig S3) and by Step-4 estimate adjustment approach (see Supplemental Methods 1.1), which follows either the default approach (denoted Poisson), is tailored to errors predicted under an internally-simulated sibship distribution directly approximating the sample data (denoted Tailored), or displays error with no adjustment made to penetrance estimates (denoted No correction). Panel rows stratify estimates according to the disease state combination from which $R(X)^{obs}$ is defined; F = familial, S = sporadic, U = unaffected, and A = affected - state X is the first state named.

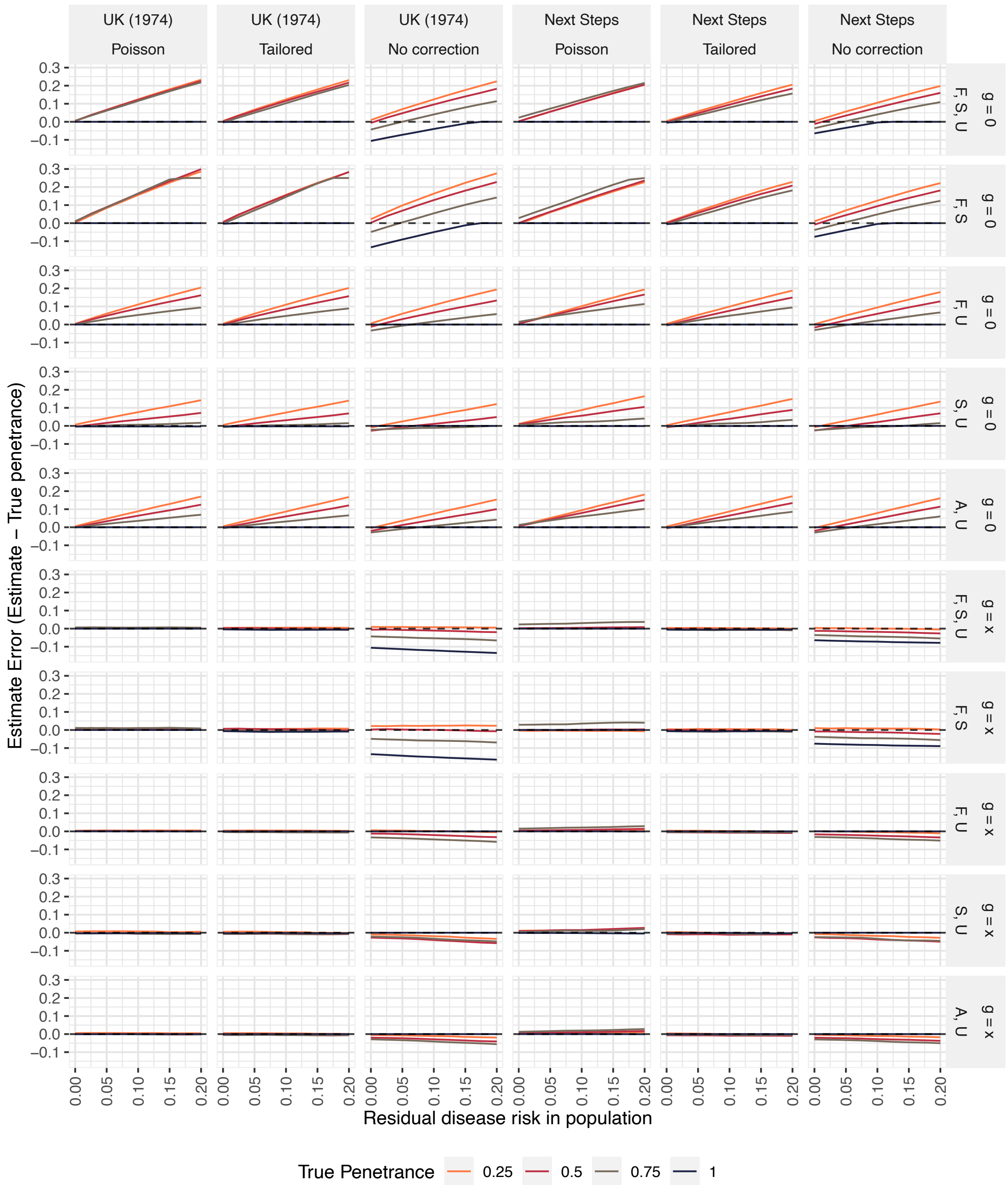

Fig S8. Error in penetrance estimates according to magnitude of disease risk g for people not harbouring the variant.

Note: Zero indicates a perfect penetrance estimate, positive values indicate overestimation and negative values underestimation. Plot lines represent true penetrance values. The x-axis indicates the probability of developing disease for people not harbouring the variant (g). *Panel columns stratify by population simulated (see Fig S3) and by Step-4 adjustment approach (see Supplemental Methods 1.1), which follows either the default approach (denoted Poisson), is tailored to errors predicted under an internally-simulated sibship distribution directly approximating the sample data (denoted Tailored) or displays error with no adjustment made to penetrance estimates (denoted No correction). Panel rows stratify*

estimates firstly *according to whether penetrance estimates account for g;* $g = 0$ *rows estimate penetrance under the assumption that people not harbouring the variant do not develop disease;* $g = x$ *rows make penetrance estimates when risk g is estimated accurately according to x-axis values. Secondly, they stratify by the disease state combination from which* $R(X)^{obs}$ *is defined; F = familial, S = sporadic, U = unaffected, and A = affected - state X is the first state named.*

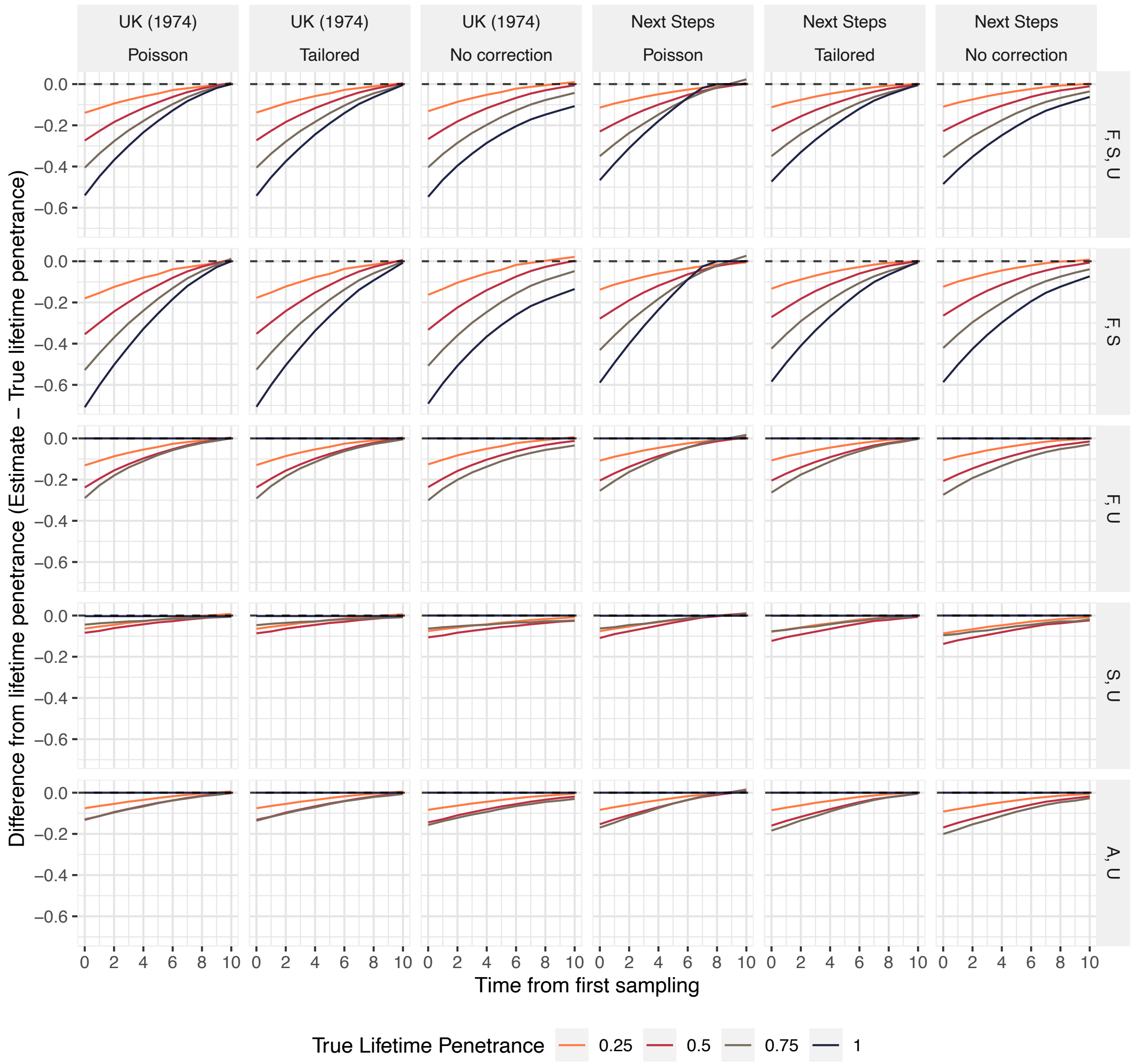

Fig S9. Penetrance according to age of sampling across only families harbouring a variant of lifetime penetrance f.

Note: Zero indicates a perfect estimate of lifetime penetrance, positive values indicate overestimation and negative values underestimation. Plot lines represent true lifetime penetrance values. The x-axis indicates sampling across time from time 0 which is when the youngest sibling reaches the age at which disease may first onset and 10 is the point at which this sib (and therefore all family members) have reached the full lifetime penetrance of disease. Panel columns stratify by population simulated (see Fig S3) and by Step-4 adjustment approach (see Supplemental Methods 1.1), which follows either the default approach (denoted Poisson), is tailored to errors predicted under an internally-simulated sibship distribution directly approximating the sample data (denoted Tailored) or displays error with no adjustment made to penetrance estimates (denoted No correction). Panel rows stratify estimates according to the disease state combination from which $R(X)^{obs}$ is defined; F = familial, S = sporadic, U = unaffected, and A = affected - state X is the first state named.

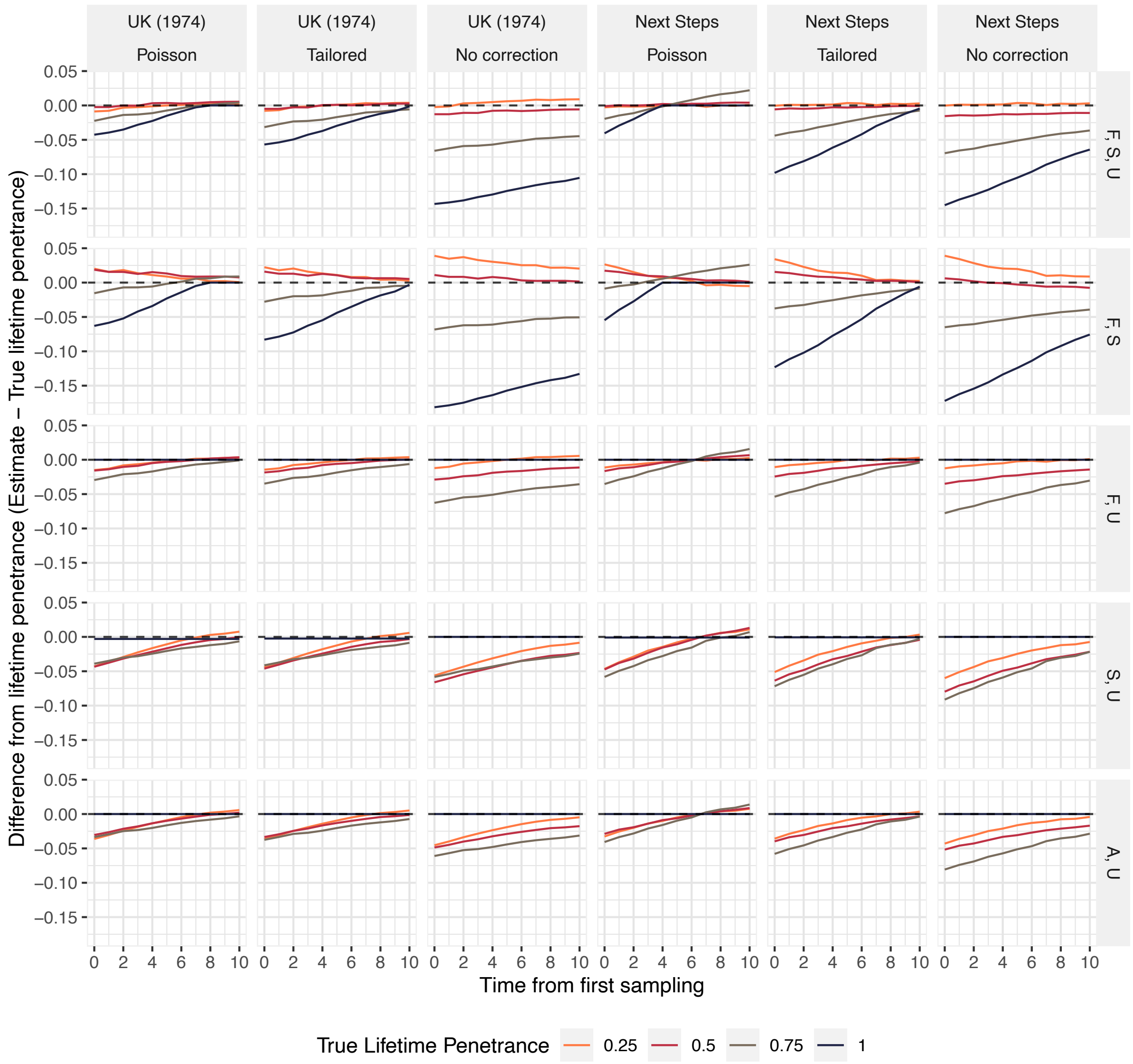

Fig S10. Error in lifetime penetrance estimates according to age of sampling based on variant frequency estimates across a wider disease cohort with equal age of onset variability.

Note: In this simulation equal onset variability is observed. Zero indicates a perfect estimate of lifetime penetrance, positive values indicate overestimation and negative values underestimation. Plot lines represent true lifetime penetrance values. The x-axis indicates time of sampling t between $t = 0$, the final age before the youngest sibling an age where disease may first onset, and $t = 10$, the point at which all family members have reached the maximum age for disease onset. Panel columns stratify by population simulated (see Fig S3) and by Step 4 adjustment approach (see Supplemental Methods 1.1), which follows either the default approach (denoted Poisson), is tailored to errors predicted under an internally-simulated sibship distribution directly approximating the sample data (denoted Tailored) or displays error with no adjustment made to penetrance estimates (denoted No correction). Panel rows stratify estimates according to the disease state combination from which $R(X)^{obs}$ is defined; F = familial, S = sporadic, U = unaffected, and A = affected – state X is the first state named.

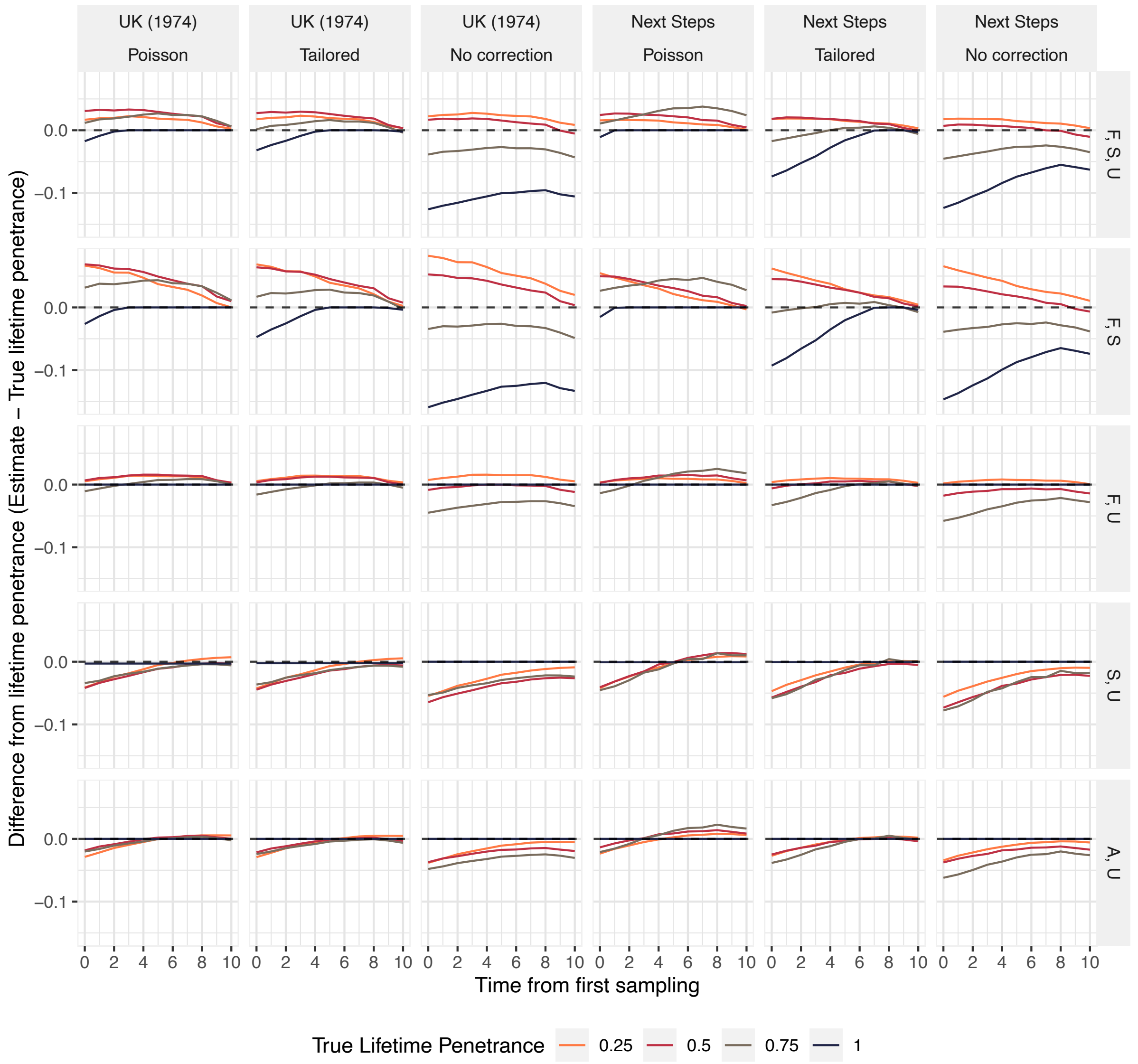

Fig S11. Error in lifetime penetrance estimates according to age of sampling based on variant frequency estimates across a wider disease cohort when age of onset density is more compressed among people harbouring the tested variant.

Note: Equal onset variability (see Supplementary methods 1.2.2) is not observed; the disease onset window is 1.3 times shorter for people harbouring the tested variant than people without the variant. Zero indicates a perfect estimate of lifetime penetrance, positive values indicate overestimation and negative values underestimation. Plot lines represent true lifetime penetrance values. The x-axis indicates time of sampling t between $t = 0$, the final age before the youngest sibling an age where disease may first onset, and $t = 10$, the point at which all family members have reached the maximum age for disease onset. Panel columns stratify by population simulated (see Fig S3) and by Step-4 adjustment approach (see Supplemental Methods 1.1), which follows either the default approach (denoted Poisson), is tailored to errors predicted under an internally-simulated sibship distribution directly approximating the sample data (denoted Tailored) or displays error with no adjustment made to penetrance estimates (denoted No correction). Panel rows stratify estimates according to the disease state combination from which $R(X)^{obs}$ is defined; F = familial, S = sporadic, U = unaffected, and A = affected - state X is the first state named.

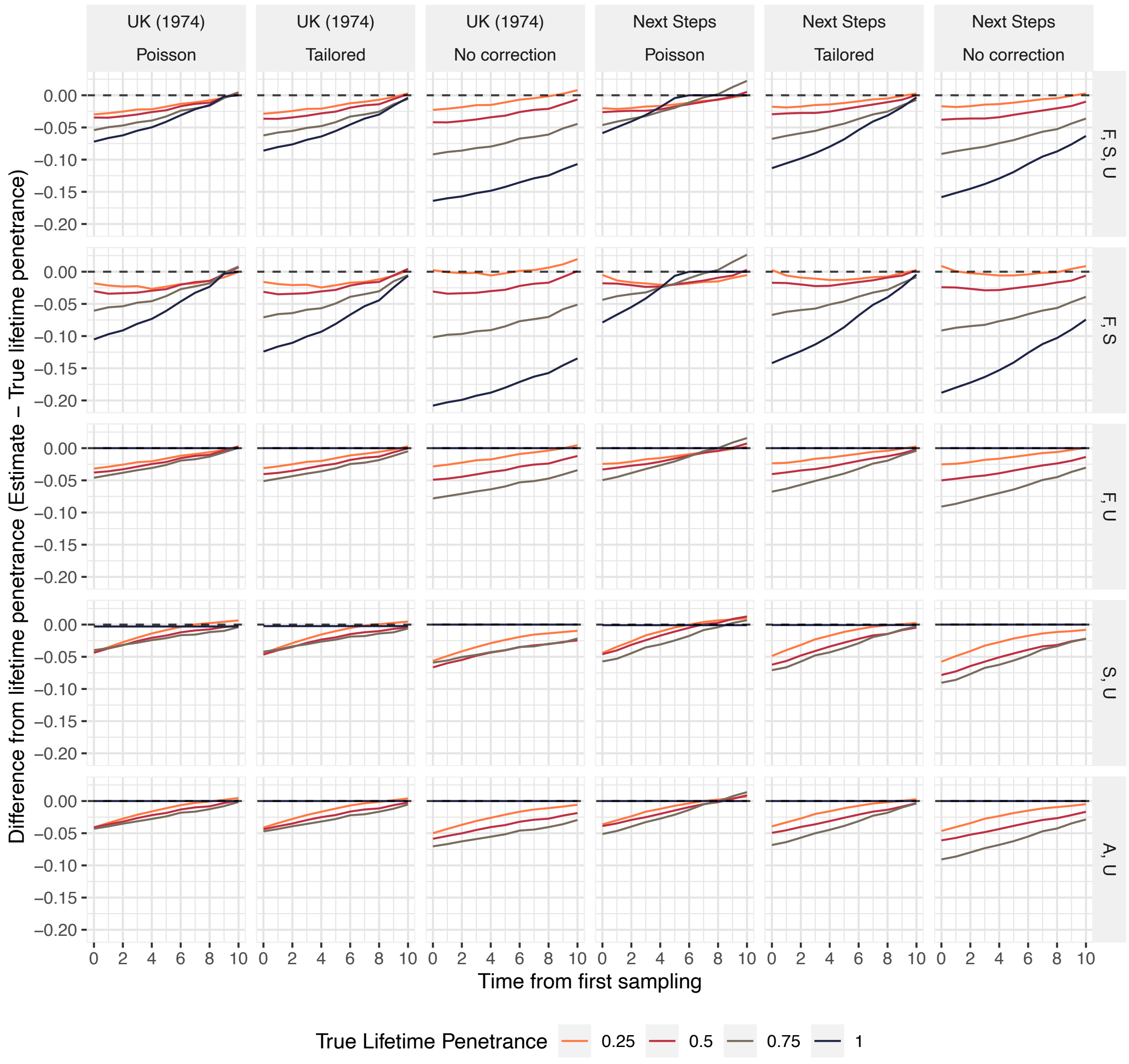

Fig S12. Error in lifetime penetrance estimates according to age of sampling based on variant frequency estimates across a wider disease cohort when age of onset density is less compressed among people harbouring the tested variant.

Note: Equal onset variability (see Supplementary methods 1.2.2) is not observed; the disease onset density in people harbouring the tested variant is 0.77 times that of people without the variant. Zero indicates a perfect estimate of lifetime penetrance, positive values indicate overestimation and negative values underestimation. Plot lines represent true lifetime penetrance values. The x-axis indicates time of sampling t between $t = 0$, the final age before the youngest sibling an age where disease may first onset, and $t = 10$, the point at which all family members have reached the maximum age for disease onset. Panel columns stratify by population simulated (see Fig S3) and by Step 4 adjustment approach (see Supplemental Methods 1.1), which follows either the default approach (denoted Poisson), is tailored to errors predicted under an internally-simulated sibship distribution directly approximating the sample data (denoted Tailored) or displays error with no adjustment made to penetrance estimates (denoted No correction). Panel rows stratify estimates according to the disease state combination from which $R(X)^{obs}$ is defined; F = familial, S = sporadic, U = unaffected, and A = affected - state X is the first state named.

3. Supplemental Tables

3.1.

Table S1. Sample characteristics and calculation of N for data applied in case study 1.

Joint population[a]		Population sampled	Number of unrelated people sampled [16]				Percentage of joint population	Calculation of sibship size (N)		
			Sporadic	Familial	Unaffected	Total		World region[b]	Weighted TFR estimate[c]	TFR Estimate for region[d]
European ancestry sample	North European	North American (white)	2606	1450	4934	8990	50.20%	North America	0.856381708	1.706
		British	1145	192	1786	3123	17.44%	United Kingdom	0.292961081	1.68
		German and Austrian	803	231	436	1470	8.21%	Germany	0.128868167	1.57
		Norwegian	371	64	572	1007	5.62%	Norway	0.08771679	1.56
		Australian	578	252	0	830	4.63%	Australia	0.080641018	1.74
		French	300	174	348	822	4.59%	France	0.086289575	1.88
		Swedish	200	127	200	527	2.94%	Sweden	0.05179072	1.76
		Irish	236	35	212	483	2.70%	Ireland	0.04719694	1.75
		Polish	153	21	190	364	2.03%	Poland	0.029674465	1.46
		Russian	157	10	126	293	1.64%	Russian Federation	0.025685968	1.57
		Total	6549	2556	8804	17909	100%	-	-	1.687206433[d]
	South European	Italian and Sardinian	2516	633	1040	4189	52.45%	Italy	0.676660406	1.29
		Spanish	806	283	544	1633	20.45%	Spain	0.257648385	1.26
		Basque	117	41	425	583	7.30%	Spain	0.091983471	1.26
		Portuguese	317	85	100	502	6.29%	Portugal	0.089261207	1.42
		Cretan	174	92	0	266	3.33%	Greece	0.044966191	1.35

		Serbian	47	51	161	259	3.24%	Serbia	0.048323316	1.49
		Greek	235	0	0	235	2.94%	Greece	0.03972577	1.35
		Chilean	137	29	153	319	3.99%	Chile	0.065869146	1.649
		Total	4349	1214	2423	7986	100%	-	-	1.314437891[d]
	All European ancestry	North European	6549	2556	8804	17,909	69.16%	-	1.166873142	1.687206433[d]
		South European	4349	1214	2423	7986	30.84%	-	0.405371732	1.314437891[d]
		Total	10,898	3770	11,227	25,895	100%	-	-	1.572244874[d]
Global ancestries sample		Chinese	1360	973	938	3271	9.55%	China	0.161344638	1.69
		Japanese	526	60	372	958	2.80%	Japan	0.039704629	1.42
		Korean	436	17	0	453	1.32%	Korean Republic	0.012917547	0.977
		Indian	718	82	1200	2000	5.84%	India	0.12970638	2.222
		North African Arabs	56	143	739	938	2.74%	Middle East and North Africa	0.076902749	2.809
		Ashkenazi Jews	259	78	410	747	2.18%	North America	0.037195202	1.706
		All European ancestry	10,898	3770	11,227	25,895	75.58%	-	1.188292598	1.572244874[d]
		Total	14,253	5,123	14,886	34,262	100%	-	-	1.64606374[d]

[a] *Populations sampled were assigned to joint ancestry regions based on the ancestry group most frequent among people from that population*
[b] *Total Fertility Rate (TFR) estimates for each individual region drawn from the World Bank database [12]: these estimates were assigned to each population using the region defined in the World Bank database that appeared most representative. Where a sampled population features more than one named country, TFR was defined by the country with the larger population. For the Ashkenazi Jewish and Basque populations, estimates were assigned based on the world regions for which their populations are largest.* [c] *Each weighted TFR value is calculated as:* $TFR\ estimate\ for\ region \times percentage\ of\ joint\ population$; [d] *Marked TFR estimates were derived by summation of all* weighted TFR estimates attributed to that region.

3.2.

Table S2. Penetrance estimation of the LRRK2 p.Gly2019Ser variant for Parkinson's Disease across populations sampled in case study 1.

	Sample	Variant frequency in state [16] (variant count / sample size)			Ave. sibship size[a]	Residual disease risk[b]	States modelled [c]	Familial disease rate among those harbouring the variant across states modelled (95% CI)	Penetrance (95% CI)[d]	
		familial	sporadic	unaffected					Assuming no residual disease risk	Accounting for residual disease risk
Individual population estimates	North American (white)	0.0310 (45/1450)	0.00998 (26/2606)	2.027×10^{-4} (1/4934)	1.706	0.0267	F, S	0.267 (0.174, 0.361)	0.436 (0.277, 0.596)	0.389 (0.23, 0.551)
	Italian and Sardinian	0.0411 (26/633)	0.0147 (37/2516)	9.615×10^{-4} (1/1040)	1.29	0.0266	F, S	0.247 (0.155, 0.339)	0.502 (0.311, 0.694)	0.447 (0.255, 0.641)
	Spanish	0.0495 (14/283)	0.0273 (22/806)	0 (0/544)	1.26	0.0262	F, S	0.175 (0.080, 0.270)	0.361 (0.159, 0.562)	0.304 (0.107, 0.506)
	Portuguese	0.141 (12/85)	0.0410 (13/317)	0 (0/100)	1.420	0.0257	F, S	0.288 (0.135, 0.441)	0.544 (0.246, 0.852)	0.494 (0.197, 0.804)
	North African Arabs	0.3566 (51/143)	0.3929 (22/56)	0.00541 (4/739)	2.809	0.0168	F,S,U	0.064 (0.036, 0.092)	0.185 (0.135, 0.227)	0.166 (0.116, 0.208)
							F, S	0.096 (0.062, 0.130)	0.097 (0.060, 0.135)	0.075 (0.037, 0.113)
							F,U	0.161 (0.026, 0.297)	0.244 (0.101, 0.333)	0.226 (0.081, 0.316)
							S,U	0.643 (0.407, 0.880)[d]	0.513 (0.254, 0.857)	0.506 (0.241, 0.856)
	Ashkenazi Jews	0.282 (22/78)	0.0965 (25/259)	0.00976 (4/410)	1.706	0.0242	F,S,U	0.063 (0.012, 0.115)	0.254 (0.103, 0.355)	0.223 (0.072, 0.323)
							F, S	0.255 (0.158, 0.353)	0.416 (0.249, 0.582)	0.373 (0.207, 0.541)
							F,U	0.078 (0.003, 0.152)	0.232 (0.050, 0.317)	0.203 (0.019, 0.289)
							S,U	0.197 (0.032, 0.363) [d]	0.127 (0.018, 0.263)	0.106 (0.001, 0.246)
Joint population estimates	North European ancestry	0.0274 (70/2556)	0.00809 (53/6549)	1.136×10^{-4} (1/8804)	1.687	0.0268	F, S	0.284 (0.212, 0.356)	0.469 (0.346, 0.592)	0.422 (0.298, 0.546)
	South European ancestry	0.0461 (56/1214)	0.0177 (77/4349)	4.127×10^{-4} (1/2423)	1.314	0.0265	F, S	0.234 (0.173, 0.295)	0.468 (0.343, 0.593)	0.412 (0.288, 0.539)
	All European ancestry	0.0334 (126/3770)	0.0119 (130/10898)	1.781×10^{-4} (2/11227)	1.572	0.0267	F, S, U	0.170 (0.092, 0.249)	0.468 (0.328, 0.587)	0.432 (0.293, 0.552)
							F, S	0.247 (0.202, 0.292)	0.429 (0.348, 0.509)	0.379 (0.299, 0.461)

							F, U	0.354 (0.034, 0.673)	0.494 (0.167, 0.709)	0.466 (0.133, 0.69)
							S, U	0.625 (0.297, 0.952) [e]	0.547 (0.212, 0.950)	0.538 (0.191, 0.95)
				4.677x10^{-4} (10/21383) Ref. 17	1.572	0.0267	F, S, U [f]	0.113 (0.071, 0.155)	0.37 (0.285, 0.443)	0.334 (0.249, 0.408)
							F, S	0.247 (0.202, 0.292)	0.429 (0.348, 0.509)	0.379 (0.299, 0.461)
							F, U [f]	0.172 (0.081, 0.264)	0.35 (0.247, 0.428)	0.32 (0.215, 0.399)
							S, U [f]	0.388 (0.235, 0.541) [e]	0.293 (0.161, 0.45)	0.275 (0.138, 0.438)
	Total Worldwide	0.03923 (201/5123)	0.01255 (179/14253)	7.389x10^{-4} (11/14886)	1.646	0.0266	F, S, U	0.098 (0.059, 0.137)	0.332 (0.251, 0.402)	0.297 (0.216, 0.366)
							F, S	0.268 (0.229, 0.307)	0.450 (0.382, 0.517)	0.402 (0.334, 0.47)
							F, U	0.134 (0.064, 0.204)	0.304 (0.216, 0.370)	0.273 (0.183, 0.341)
							S, U	0.297 (0.170, 0.424) [e]	0.209 (0.110, 0.324)	0.188 (0.086, 0.307)
				5.485x10^{-4} (30/54699) Ref. 17	1.646	0.0266	F, S, U [f]	0.117 (0.089, 0.145)	0.368 (0.315, 0.416)	0.332 (0.28, 0.38)
							F, S	0.268 (0.229, 0.307)	0.45 (0.382, 0.517)	0.402 (0.334, 0.47)
							F, U [f]	0.173 (0.118, 0.227)	0.342 (0.287, 0.389)	0.312 (0.256, 0.36)
							S, U [f]	0.363 (0.273, 0.452) [e]	0.266 (0.189, 0.351)	0.248 (0.168, 0.336)

Population specific penetrance estimates were made only for those with at least 5 people harbouring LRRK2 p.Gly2019Ser in both the familial and sporadic states. Lifetime disease risk, $P(A)^{pop}$, was 1/37 (0.027) in all calculations; the proportions familial, $P(F|A)$, and sporadic, $P(S|A)$, were respectively 0.105 and 0.895. The familial and sporadic states were modelled in all included populations. Penetrance was also modelled using the unaffected state in only the North African Arabic and Ashkenazi Jewish populations because the variant was sparse in all control samples – occurring predominantly in these two groups. As the variant count was low in the unaffected state for all joint population estimates, we conducted estimates using all states modelled for the All European ancestry and Total Worldwide samples only; these analyses were conducted using the main dataset [16] and repeated with variant frequency for the unaffected state estimated using the larger sample of the gnomAD v2.1.1 (controls) database [17].
[a]Estimated using Total Fertility Rates described for each population in Table S1; [b] Derived per equations 9-11; [c]F=familial, S=sporadic, U=unaffected (controls); [d]Step 4 penetrance estimates are shown; [e]Rate of sporadic disease has been calculated here because the familial state is not represented; [f]Unaffected variant frequency estimated from the gnomAD v2.1.1 (controls) sample, using the full sample for total worldwide and the European (non-Finnish) sample for all European ancestry; 95% CI = confidence interval.

3.3.

Table S3. Penetrance estimation for heterozygous inheritance of widely-described SOD1 variants.

SOD1 variant	Variant frequency in state (variant count[a] / sample size)			Lifetime risk of disease [18]	Proportion familial[b]	Average sibship size[c]	Residual disease risk[d]	States modelled[e]	Familial disease rate among those harbouring the variant across states modelled (95% confidence interval)	Penetrance (95% Confidence interval)[h]	
	familial [19]	sporadic [14]	unaffected [17]							Assuming no residual disease risk	Accounting for residual disease risk
p.Ala5Val	0.006222 (7/1125)	0.000229 (1/4366)	-	0.0025	0.050	1.543	0.0025	F, S	0.588 (0.081, 1[f])	1 (0.133, 1)	1 (0.128, 1)
p.Asp91Ala[g]	-	0.000916 (4/4366)	0.00137 (33/24,143)	0.0025	0.050	1.543	0.0025	S, U	1.59×10^{-3} (0.000, 0.003)[h]	8.98×10^{-5} (0, 0.001)	0.000 (0, 0)
p.Ile114Thr	0.01491 (17/1140)	0.001374 (6/4366)	-	0.0025	0.050	1.543	0.0025	F, S	0.364 (0.149, 0.578)	0.648 (0.255, 1)	0.644 (0.25, 1)

Variant frequencies are estimated using the ALS Variant Server [19] for the familial state, the ProjectMinE database [14] for the sporadic state and the European (non-Finnish) population of the gnomAD v2.1.1 (control) database [17] for the unaffected state.
[a]The number of people heterozygous for the tested variants; sample size = the number of people sequenced for variants at this locus; [b]Proportion sporadic is defined as 1 – proportion familial ($P(S|A) = 1 - P(F|A)$); [c]Estimated based on Total Fertility Rates for the European Union region in 2018 (Ref. 12); [d]Derived per equations 9-11, letting unaffected variant frequency equal 0 for p.Ala5Val and p.Ile114Thr, and familial variant frequency equal 0 for p.Asp91Ala; [e]F=familial, S=sporadic, U=unaffected; [f]The familial disease rate estimate is truncated to 1 as the upper 95% confidence interval bound exceeds the highest possible frequency; [g]p.Asp91Ala is most frequently associated with autosomal recessive ALS presentations, we have modelled the penetrance of its autosomal dominant form only; [h]Rate of sporadic disease has been calculated here because the familial state is not represented – no occurrences of the SOD1 p.Asp91Ala variant are reported in the ALS Variant Server. [h]Step 4 penetrance estimates are shown.

3.4.

Table S4. Estimation of the incidence of amyotrophic lateral sclerosis relative to frontotemporal dementia among people of European ancestry who harbour the pathogenic hexanucleotide GGGGCC repeat expansion of the C9orf72 gene (C9orf72^{RE}).

		Phenotype		Mathematical notation
		**ALS**	**FTD**	
Published data	Lifetime risk (1/N)	1/400 [Ref. 18]	1/742 [Ref. 20]	A
	Familial disease rate (freq.)	0.05 [Refs. 21,22]	0.30 [Ref. 22]	B
	C9orf72^{RE} rate in familial state (freq.)	0.32 [Ref. 23]	0.248 [Ref. 24]	C
	C9orf72^{RE} rate in sporadic state (freq.)	0.05 [Ref. 23]	0.060 [Ref. 24]	D
Estimated value$^{\Delta}$	Overall *C9orf72*^{RE} rate (freq.)	0.064	0.116	E[a]
	Rate of *C9orf72*^{RE} and phenotype in population (freq.)	1.588x10^{-4}	1.568x10^{-4}	F[b]
	Incidence relative to FTD among people harbouring *C9orf72*^{RE}	1.012	-	G[c]

Calculations are shown with respect to mathematical notation assigned to each row:

[a] $E = (C \times B) + (D \times (1 - B))$;

[b] $F = E \times A$;

[c] $G = F_{ALS}/F_{FTD}$.

3.5.

Table S5. Comparison of unadjusted penetrance estimates derived for the case studies presented in Table 2 between the lookup table and maximum-likelihood approaches.

Case study	Data subset	Residual disease risk[a]	States modelled[b]	Unadjusted penetrance estimates (95% Confidence interval)[a]		Adjusted penetrance (95% Confidence interval)[a,d]
				Lookup approach	Non-Linear Minimisation[c]	
LRRK2 p.G2019S for PD[16]	European ancestry	0.0267	F, S, U	0.338 (0.256, 0.407)	0.338 (0.256, 0.407)	0.334 (0.249, 0.408)
			F, S	0.393 (0.319, 0.464)	0.393 (0.319, 0.464)	0.379 (0.299, 0.461)
			F, U	0.319 (0.218, 0.393)	0.319 (0.218, 0.393)	0.32 (0.215, 0.399)
			S, U	0.257 (0.129, 0.414)	0.257 (0.129, 0.414)	0.275 (0.138, 0.438)
BMPR2 variants for PAH	All variants[25]	0.0401	F, S	0.33 (0.289, 0.369)	0.33 (0.289, 0.369)	0.309 (0.267, 0.352)
	All variants[26]	0.0388	F, S	0.237 (0.144, 0.326)	0.236 (0.144, 0.326)	0.212 (0.121, 0.305)
	Small variants[26]	0.0413	F, S	0.25 (0.133, 0.361)	0.249 (0.133, 0.361)	0.225 (0.11, 0.343)
	Large variants[26]	0.0475	F, S	0.162 (0, 0.363)	0.162 (0, 0.363)	0.138 (0, 0.345)
SOD1 variants for ALS[27]	Asian	0.00243	F, S	0.746 (0.626, 0.861)	0.746 (0.626, 0.861)	0.826 (0.661, 1)
	European	0.00245	F, S	0.656 (0.49, 0.809)	0.656 (0.49, 0.809)	0.701 (0.491, 0.926)
C9orf72[RE] for ALS[23]	Asian	0.00247	F, S	0.278 (0.019, 0.511)	0.278 (0.019, 0.511)	0.258 (0.011, 0.518)
	European	0.00234	F, S	0.445 (0.373, 0.514)	0.445 (0.373, 0.514)	0.439 (0.358, 0.52)

[a]All penetrance estimates take into account residual risk g, calculated in accordance with equations 9-11; [b]*F=familial, S=sporadic, U=unaffected (controls);* [c]*Approach is described in Supplemental Methods 1.2.1;* [d]*Adjusted penetrance estimates were derived from unadjusted lookup approach estimate, but are representative of both methods.*

PD = Parkinson's disease, PAH = pulmonary arterial hypertension, ALS = amyotrophic lateral sclerosis, C9orf72[RE] *= the pathogenic C9orf72 GGGGCC hexanucleotide repeat expansion.*

3.6.

Table S6. *Direction of change in R(X)^obs and penetrance estimates according to increases in variant frequency and weighting factor inputs.*

Parameter (Notation)	States modelled[a]				
	F, S, U	F, S	F, U	S, U	A, U
Variant frequency in familial state (M_F)	↑	↑	↑	-	-
Variant frequency in sporadic state (M_S)	↓	↓	-	↑	-
Variant frequency in unaffected state (M_U)	↓	-	↓	↓	↓
Variant frequency in affected state (M_A)	-	-	-	-	↑
Familial disease rate ($P(F\|A)$)	↑	↑	↑	↓	-
Probability of a person in population being affected ($P(A)^{pop}$)	↑	-	↑	↑	↑

[a] *F=familial, S=sporadic, U=unaffected (controls), A= Affected.*

4. Supplemental References

1. Office for National Statistics. Childbearing for women born in different years. 2020. Available from: https://www.ons.gov.uk/peoplepopulationandcommunity/birthsdeathsandmarriages/conceptionandfertilityrates/datasets/childbearingforwomenbornindifferentyearsreferencetable
2. Hughes I, Hase T. Measurements and their uncertainties: a practical guide to modern error analysis. Oxford: Oxford University Press; 2010.
3. R Core Team. R: A language and environment for statistical computing. 2021. R Foundation for Statistical Computing, Vienna, Austria. Available from: https://www.R-project.org/
4. Spargo TP, Opie-Martin S, Bowles H, Lewis CM, Iacoangeli A, Al-Chalabi A. ADPenetrance. 2022. GitHub. Available from: https://github.com/ThomasPSpargo/adpenetrance
5. Wickham H. ggplot2: Elegant Graphics for Data Analysis: Springer-Verlag New York; 2016.
6. Wickham H. Reshaping Data with the reshape Package. J Stat Softw. 2007; 21(12):1-20. doi:10.18637/jss.v021.i12
7. Wickham H. The Split-Apply-Combine Strategy for Data Analysis. J Stat Softw. 2011; 40(1):1-29. doi:10.18637/jss.v040.i01
8. Kirmeyer SE, Hamilton BE. Childbearing Differences Among Three Generations of U.S. Women. National Center for Health Statistics; 2011. Available from: https://www.cdc.gov/nchs/data/databriefs/db68.pdf
9. Sheppard P, Monden C. When does family size matter? Sibship size, socioeconomic status and education in England. Evol Hum Sci. 2020; 2, e51:1-21. doi:10.1017/ehs.2020.54
10. Al-Chalabi A, Lewis CM. Modelling the Effects of Penetrance and Family Size on Rates of Sporadic and Familial Disease. Hum Hered. 2011; 71(4):281-8. doi:10.1159/000330167
11. Chang W, Cheng J, Allaire J, et al. shiny: Web Application Framework for R. 2022. R package version 1.7.3. Available from: https://CRAN.R-project.org/package=shiny
12. Fertility rate, total (births per woman) [Internet]. 2020. Available from: https://databank.worldbank.org/reports.aspx?source=2&series=SP.DYN.TFRT.IN
13. Project MinE ALS Sequencing Consortium. Project MinE: study design and pilot analyses of a large-scale whole-genome sequencing study in amyotrophic lateral sclerosis. Eur J Hum Genet. 2018; 26(10):1537-46. doi:10.1038/s41431-018-0177-4
14. van der Spek RAA, van Rheenen W, Pulit SL, Kenna KP, van den Berg LH, Veldink JH. The project MinE databrowser: bringing large-scale whole-genome sequencing in ALS to researchers and the public. Amyotroph Lateral Scler Frontotemporal Degener. 2019; 20:432-40. doi:10.1080/21678421.2019.1606244
15. Opie-Martin S, Iacoangeli A, Topp SD, et al. The *SOD1*-mediated ALS phenotype shows a decoupling between age of symptom onset and disease duration. Nat Commun. 2022; 13(1):6901. doi:10.1038/s41467-022-34620-y
16. Healy DG, Falchi M, O'Sullivan SS, et al. Phenotype, genotype, and worldwide genetic penetrance of *LRRK2*-associated Parkinson's disease: a case-control study. Lancet Neurol. 2008; 7(7):583-90. doi:10.1016/S1474-4422(08)70117-0
17. Karczewski KJ, Francioli LC, Tiao G, et al. The mutational constraint spectrum quantified from variation in 141,456 humans. Nature. 2020; 581(7809):434-43. doi:10.1038/s41586-020-2308-7
18. Alonso A, Logroscino G, Jick SS, Hernán MA. Incidence and lifetime risk of motor neuron disease in the United Kingdom: a population-based study. Eur J Neurol. 2009; 16(6):745-51. doi:10.1111/j.1468-1331.2009.02586.x

19. ALS Variant Server [Internet]. [cited 02/2021]. Available from: http://als.umassmed.edu/.
20. Coyle-Gilchrist ITS, Dick KM, Patterson K, et al. Prevalence, characteristics, and survival of frontotemporal lobar degeneration syndromes. Neurology. 2016; 86(18):1736. doi:10.1212/WNL.0000000000002638
21. Byrne S, Walsh C, Lynch C, et al. Rate of familial amyotrophic lateral sclerosis: a systematic review and meta-analysis. J Neurol Neurosurg Psychiatry. 2011; 82(6):623-7. doi:10.1136/jnnp.2010.224501
22. Turner MR, Al-Chalabi A, Chio A, et al. Genetic screening in sporadic ALS and FTD. J Neurol Neurosurg Psychiatry. 2017; 88(12):1042-4. doi:10.1136/jnnp-2017-315995
23. Marogianni C, Rikos D, Provatas A, et al. The role of C9orf72 in neurodegenerative disorders: a systematic review, an updated meta-analysis, and the creation of an online database. Neurobiol Aging. 2019:1.e-.e10. doi:10.1016/j.neurobiolaging.2019.04.012
24. Majounie E, Renton AE, Mok K, et al. Frequency of the *C9orf72* hexanucleotide repeat expansion in patients with amyotrophic lateral sclerosis and frontotemporal dementia: a cross-sectional study. Lancet Neurol. 2012; 11(4):323-30. doi:10.1016/s1474-4422(12)70043-1
25. Evans JDW, Girerd B, Montani D, et al. *BMPR2* mutations and survival in pulmonary arterial hypertension: an individual participant data meta-analysis. Lancet Respir Med. 2016; 4(2):129-37. doi:10.1016/S2213-2600(15)00544-5
26. Aldred MA, Vijayakrishnan J, James V, et al. *BMPR2* gene rearrangements account for a significant proportion of mutations in familial and idiopathic pulmonary arterial hypertension. Hum Mutat. 2006; 27(2):212-3. doi:10.1002/humu.9398
27. Zou Z-Y, Zhou Z-R, Che C-H, Liu C-Y, He R-L, Huang H-P. Genetic epidemiology of amyotrophic lateral sclerosis: a systematic review and meta-analysis. J Neurol Neurosurg Psychiatry 2017; 88:540-9. doi:10.1136/jnnp-2016-315018
